# Neurofunctional MOF nanoparticles integrated with extracellular matrix hydrogel for neuro-Vascularized bone regeneration

**DOI:** 10.1016/j.mtbio.2025.102541

**Published:** 2025-11-12

**Authors:** Ning Sheng, Runze Yang, Jie Wang, Wenting Wu, Man Zhe, Qing-Yi Zhang, Rong Nie, Long Chen, Fei Xing, Li Sun

**Affiliations:** a Department of Orthopedics, Guizhou Provincial People's Hospital, Guiyang, 550000, Guizhou, People's Republic of China; b Department of Pediatric Surgery, Division of Orthopedic Surgery, Orthopedic Research Institute, Laboratory of Stem Cell and Tissue Engineering, State Key Laboratory of Biotherapy, West China School of Medicine, West China Hospital, Sichuan University, Chengdu, 610041, People's Republic of China; c Department of Orthopedics, Sichuan Provincial People's Hospital, University of Electronic Science and Technology of China, Chengdu, 610072, People's Republic of China; d Department of Foot and Ankle Surgery, Honghui Hospital of Xi'an Jiaotong University, Xi'an City, Shaanxi, 710054, People's Republic of China; e Animal Experiment Center, West China Hospital, Sichuan University, Chengdu, 610041, People's Republic of China; f Department of Orthopedic Surgery and Orthopedic Research Institute, Laboratory of Stem Cell and Tissue Engineering, State Key Laboratory of Biotherapy, West China Hospital, Sichuan University, Chengdu, 610041, People's Republic of China; g Sports Medicine Center, Department of Orthopedic Surgery/Orthopedic Research Institute, West China Hospital, Sichuan University, Chengdu, Sichuan, 610064, People's Republic of China

**Keywords:** Extracellular matrix, Hydrogel, Zn-MOF, Substance P, Neuro-vascularized, Bone regeneration

## Abstract

The neural and vascular systems are the basic elements of bone. Neurovascular networks are widely distributed throughout the periosteum, cortical bone, and cancellous bone, which is highly significant for bone regeneration and remodeling. Reconstruction of the neurovascular network has been proposed as a therapeutic approach for bone defects. Here, a neuropeptide substance P-loaded nano-MOF combined with a double network hydrogel (PM@PS hydrogel) was applied to the bone regeneration environment to promote neuroangiogenesis by modulating Schwann cells (SCs) and human umbilical vein endothelial cells (HUVECs). It further enhances the osteogenic differentiation of bone marrow stromal cells (BMSCs) by activating the Wnt signaling pathway. In vivo experiments also demonstrate that functionalized hydrogels promote nerve ingrowth and vascularization, leading to neurovascular-driven bone regeneration. These findings emphasize the important role of the neurovascular network in bone regeneration and provide a new therapeutic strategy to facilitate the repair of critical-size bone defects.

## Highlights

• An injectable double-network hydrogel (PM@PS) derived from small intestinal submucosa extracellular matrix is designed for bone repair.
• As hydrogel degrades, it releases substance P-loaded Zn-MOF nanoparticles and ECM components for nerve repair and promoting angiogenesis.
• The hydrogel drives neurovascular-mediated bone regeneration by activating the Wnt signaling pathway to enhance osteogenesis.
• The PM@PS hydrogel demonstrated early neurogenic and angiogenic effects, effectively promoting bone formation and repair.

## Graphical abstract

**Figure:**
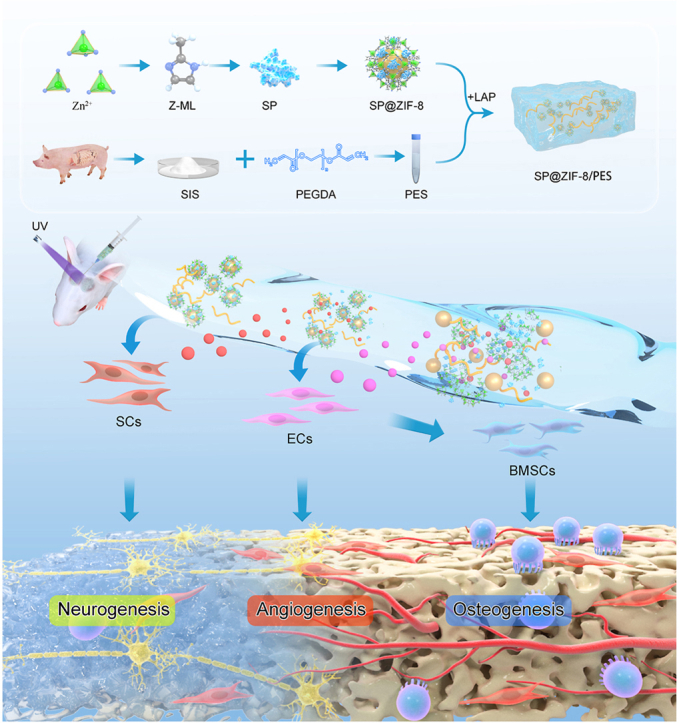

## 1 Introduction

Reconstruction for critical bone defects caused by trauma, infections, tumors, and other diseases is still a clinical challenge. As a complex, highly coordinated process, bone regeneration involves intricate molecular interactions among cytokines, stem cells, and environmental factors. Among these factors, early vascularization has been recognized as a key determinant of bone regeneration [1]. Consequently, enhancing angiogenesis has emerged as a crucial therapeutic strategy [2]. More recently, increasing attention has been paid to the nervous system in bone repair [3]. Sensory and sympathetic nerve fibers are abundantly distributed in bone tissue and contribute to repair by releasing neuropeptides and neurotransmitters [4]. Following injury, nerve fibers initiate the repair cascade within hours [5]. Neuropeptides such as calcitonin gene-related peptide (CGRP) and vasoactive intestinal peptide (VIP) have been shown to promote osteogenic differentiation by modulating the local microenvironment [[6], [7], [8]]. These findings support the concept that early regeneration of the neural network also plays a crucial role in promoting the healing of critical-size bone defects.

Substance P (SP) is a member of the tachykinin family of neuropeptides released from nociceptive sensory neurons [9]. SP is an endogenous agonist for neurokinin-1 receptor (NK1R) with which it binds with high affinity. NK1R transduces signals through the inositol triphosphate/diacylglycerol and cyclic adenosine monophosphate second messenger system to activate pERK1/2, and ultimately mediate the transcription of chemokines and cytokines [10]. SP is involved in many biological processes and is therefore considered to have great potential for repair [11]. Studies have proven that SP can promote the recruitment of endogenous stem cells and effectively enhance peripheral nerve regeneration [12,13], suggesting its potential as a therapeutic agent for innervated bone regeneration. However, to avoid rapid enzymatic degradation and achieve sustained biological effects, an appropriate delivery carrier is essential for the localized and prolonged release of SP at the defect site.

Metal-organic frameworks (MOFs) are crystalline materials composed of metal ions and organic ligands, and they have shown great promise in biomedical applications due to their tunable porosity and high drug-loading capacity. Among them, zinc-based MOF (Zn-MOF), particularly zeolitic imidazolate framework-8 (ZIF-8), have attracted significant interest due to their mild synthesis conditions, good biocompatibility, and high structural stability [[14], [15], [16]]. Moreover, zinc ions (Zn^2+^), released during ZIF-8 degradation, serve as essential cofactors in biological systems and have been demonstrated to promote angiogenesis in vitro and in vivo [17,18]. These features make Zn-MOF an ideal candidate for co-delivering SP and supporting neurovascular regeneration in bone repair [19,20].

While nanoparticles offer excellent drug delivery capabilities, they often face challenges such as rapid clearance and limited retention at the target site. In contrast, biomaterial scaffolds provide a three-dimensional architecture that supports cell adhesion, proliferation, and differentiation. An ideal scaffold for bone regeneration should possess good biocompatibility, appropriate degradation kinetics, and a porous structure to allow for vascular and nerve ingrowth [3]. Among various scaffold materials, hydrogels have emerged as attractive candidates due to their high water content, extracellular matrix (ECM)-like architecture, and adaptability to irregularly shaped defects [21,22]. In particular, small intestinal submucosa (SIS) derived hydrogels - obtained from decellularized porcine intestinal tissue - retain native ECM components and bioactive factors such as VEGF, EGF, and bFGF, which are known to support angiogenesis [[23], [24], [25]]. The decellularization process removes immunogenic cellular components, minimizing the risk of immune rejection, as demonstrated in prior studies and clinical applications. Our previous studies demonstrated that SIS-based hydrogels significantly promote angiogenesis and enhance bone repair [26,27]. Additionally, their injectable and thermosensitivity properties enable minimally invasive, in situ application, which is advantageous for clinical translation.

In this study, we developed an injectable composite hydrogel by incorporating SP-loaded Zn-MOF (PM) nanoparticles into a SIS-derived ECM matrix, followed by the addition of polyethylene glycol diacrylate (PEGDA) for crosslinking, yielding the PM@PS double network hydrogel. This multifunctional hydrogel provides sustained release of SP, Zn^2+^ and ECM component to support Schwann cell (SC) activity and neurotrophic factor secretion, and promotes endothelial cell migration and angiogenesis. Furthermore, in a co-culture conditioned medium system, composed of HUVEC and SC supernatants mixed with osteogenic induction medium, the hydrogel was shown to promote BMSC osteogenic differentiation through neurovascular modulation. In vivo experiments confirmed that the hydrogel facilitates early neurovascular network reconstruction and significantly enhances bone regeneration in a rat critical-size calvarial defect model.

## 2 Materials and methods

### 2.1 Materials

2-methylimidazole, Zinc nitrate hexahydrate (Zn(NO_3_)_2_·6H_2_O) and PEGDA were purchased from Aladdin (China), SP was purchased from MedChemExpress (USA). Calcein/PI Cell Viability/Cytotoxicity Assay Kit, Cell Counting Kit-8 (CCK-8), alkaline phosphatase (ALP), and Alizarin Red S (ARS) kits were obtained from the Beyotime Institute of Biotechnology (China). All the antibodies, including anti-CGRP, anti-β3-tubulin, anti-CD31, anti-α-SMA, anti-CD44, anti-CD105, anti-BMP-2, anti-RUNX2, anti-S100β and anti-OCN, were procured from the companies Abcam and Abclonal. Other materials can be found in Supporting Information.

### 2.2 Cell culture

Rats BMSCs were isolated from the bone marrow of Sprague-Dawley rats (2 weeks) and the cells were cultured in α-minimum essential medium (α-MEM) with 10 % fetal bovine serum (FBS) and 1 % Penicillin-Streptomycin Solution (P/S). Rat Schwann Cells (RSC 96) and Human Umbilical Vein Endothelial Cells (HUVECs) were purchased from the Chinese Academy of Science (China). RSC 96 was cultured in Dulbecco's modified Eagle's medium (DMEM) supplemented with 10 % FBS and 1 % P/S. HUVECs were cultured with F12/DMEM medium, containing 10 % FBS and 1 % P/S. All cells were incubated in a 37 °C incubator with 5 % CO_2_.

### 2.3 Synthesis and characterization of Zn-MOF and PM nanoparticles

The Zn-MOF nanoparticles were synthesized as described by Chen [28]. First, Zn(NO_3_)_2_·6H_2_O and 2-Methylimidazole were dissolved in deionized water, respectively. Second, the two solutions were mixed in the ratio of 1 : 1 and stirred at 30 °C for 10 min. The Zn-MOF nanoparticles were obtained from centrifugation (3500 rpm, 20 min), followed by washing with deionized water twice. To synthesize PM nanoparticles, 10 μL SP solution (0.05 mg/mL) was added into the 2-Methylimidazole solution before being mixed with Zn(NO_3_)_2_·6H_2_O solution.

The microstructure and average diameter of Zn-MOF and PM nanoparticles were observed by scanning electron microscopy (SEM, ZEISS GeminiSEM 300, Germany) and transmission electron microscope (TEM, JEM-2100Plus, Japan). Fourier transform infrared spectrometry (FTIR, Thermo Fisher Scientific Nicolet iS20, USA), X-ray diffraction (XRD, Rigaku MiniFlex 600, Japan), thermogravimetric analysis (TGA, PerkinElmer STA 6000, USA), and ultraviolet–visible (UV–Vis) spectroscopy (Perkin Elmer, USA) were carried out to analyze the characteristics of Zn-MOF and PM nanoparticles. To analyze the degradation rate of PM nanoparticles, nanoparticles were added to the buffer solution at pH = 7.4, pH = 6.5, and pH = 5.5, respectively. All solutions were collected at different times to measure the concentration of Zn^2+^.

### 2.4 Synthesis and characterization of hydrogels

SIS powder was fabricated as a previously described method [26]. Briefly, the tissue was subjected to mechanical scraping, enzymatic digestion (trypsin-EDTA), and detergent-based treatments (0.1 % SDS) to remove cellular components and DNA. Residual DNA content was confirmed to be < 50 ng/mg, below the threshold for immunogenicity, ensuring minimal risk of immune rejection in vivo. The details are shown in the Supporting information. To prepare the double network hydrogels, SIS powder (3 %, w/v) and PEGDA (5 %, v/v) were dissolved in a phosphate buffer solution (PBS). Photoinitiator lithium acylphosphinate salt (LAP, 0.25 %, w/v) was added to form a based hydrogel solution (PS hydrogel). SP, MOF, and PM nanoparticles were also added to synthesize the P@PS hydrogels, M@PS, and PM@PS hydrogels, respectively. In the study of Hong. et al., 100 nM SP can stimulate BMSC transmigration and proliferation [29]. Considering the encapsulation efficiency of SP and the sustained-release effect of hydrogel, we added 10 mg PM nanoparticles in 1 mL PM@PS hydrogel. To make the gel, the first network was formed at 37 °C for 5 min, and then the hydrogel was exposed to UV light for 10 min to obtain the double network PM@PS hydrogel.

The morphology of the lyophilized hydrogels was observed via SEM. The functional groups of hydrogels were tested by FTIR. The rheometer (Physica MCR302, Austria) was used to assess the viscoelastic behavior. Different hydrogels were put into PBS at 37 °C for 24 h. The original hydrogels' weight (Wo) and the change of hydrogels’ weight (Wc) were collected to calculate the swelling ratio by the following equation:

$$\text{Swelling}\;\text{ratio}\;\left(\%\right)=\frac{W_{c}}{W_{o}}\times 100\%$$

To measure the degradation behavior of hydrogels, hydrogels were submerged in PBS at 37 °C and placed in a shaker. Hydrogels were taken out at different times, washed with deionized water, then lyophilized and weighed. The weights of the initial (Wi) and remaining hydrogels at different times (Wt) were recorded, respectively. The degradation ratio was determined as the following equation:

$$\text{Degradation}\;\text{ratio}\;\left(\%\right)=\frac{W_{i}-W_{t}}{W_{i}}\times 100\%$$

The release experiment of SP was carried out in 3 mL PBS containing 1 mg hydrogels shaken at 37 °C. At a certain time, the supernatant was collected and replaced with fresh PBS. The concentration of SP was measured via enzyme-linked immunosorbent assay (ELISA) to calculate the cumulative release ratio:

$$C\text{umulative}\;\text{release}\;\text{ratio}\;\left(\%\right)=\frac{\sum \text{Wn}}{W_{1}}\times 100\%$$

Where W_n_ represents the weight of SP at each time point when the supernatants were collected, while W_1_ represents the original SP weight.

The cell proliferation of BMSCs was evaluated using the CCK-8 Assay. BMSCs were seeded in 96-well plated and co-cultured with hydrogels for 1, 2, and 3 days. Each well was rinsed with PBS. Then, the CCK-8 solution was added according to the manufacturer's instructions and incubated for 2 h. The supernatant was collected to detect the OD value. Cell activity was evaluated via live/dead cell staining at 1 and 5-day intervals. After washing with PBS, cells were incubated with calcein-AM/PI buffer for 30 min and observed by an inverted fluorescence microscope. The hemolysis test was used to evaluate the hemocompatibility of hydrogels. Briefly, fresh rabbit blood was mixed with 3.8 % sodium citrate to prepare anticoagulated whole blood. After centrifugation and washed with PBS for 3 times, the erythrocytes were resuspended with PBS (5 %, v/v). 200 μL hydrogel was added. The negative and positive controls were 200 μL PBS and 0.1 % Triton X-100, respectively. After incubating at 37 °C for 1 h, the mixed solution was centrifuged at 1000 rpm for 10 min. The absorbance value of the supernatant was recorded to calculate the hemolysis ratio as the following equation:

$$\text{Hemolysis}\;\text{ratio}\;\left(\%\right)=\frac{A_{H}-A_{N}}{A_{P}-A_{N}}\times 100\%$$

Where A_H_ represents the absorbance value of sample with hydrogels. A_N_ and A_P_ represent PBS and Triton X-100, respectively.

### 2.5 In vitro neurogenesis properties of hydrogels

The composite hydrogels were submerged in RSC 96 medium to obtain the extract. In short, the composite hydrogels (PS, P@PS, M@PS, and PM@PS) were prepared as described in Section 2.4, consisting of SIS powder (3 %, w/v), PEGDA (5 %, v/v), LAP photoinitiator (0.25 %, w/v), and, for PM@PS, 10 mg/mL SP-loaded Zn-MOF (PM) nanoparticles. To obtain the extract, 1 mg of each hydrogel was submerged in 10 mL of RSC 96 cell culture medium (DMEM supplemented with 10 % FBS and 1 % P/S) for 24 h at 37 °C. The mixture was then centrifuged at 15,000 rpm for 10 min, and the supernatant was collected as the hydrogel extract for subsequent assays. The recruitment properties of hydrogels for RSC 96 were examined using a transwell migration assay and scratch wound assay. For the transwell migration assay, RSC 96 was seeded in the upper layer of transwell inserts and the extract was added to the well of a 24-well plate. After incubation for 6 h, the cells, migrated through the permeable membrane, in the button of inserts were stained with 0.1 % (w/v) crystal violet and counted. For scratch wound assay, cells were seeded into 6-well plates. When cells were grown to confluence, a "wound" was introduced by scratching with a 500 μL pipette tip. Images were taken immediately, 24 h and 48 h.

ELISA and real-time quantitative PCR (q-PCR) were used to detect the gene expression and secretion of neurotrophic factors from RSC 96, including brain-derived neurotrophic factor (BDNF), glial cell-derived neurotrophic factor (GDNF) and nerve growth factor (NGF). Cells seeded on the surface of hydrogels for 2 days. The supernatant was collected for ELISA. For q-PCR, the cells were used to obtain the total RNA, which was reverse-transcribed into cDNA. q-PCR analysis was performed to evaluate gene transcription levels. The sequences of primers used are listed in Table S1, Supporting Information.

Immunofluorescent staining was conducted to test the protein synthesis of S100 in RSC 96. After incubation on the surface of hydrogels for 2 days, cells were fixed with paraformaldehyde (4 %, w/v), followed by permeabilized (0.25 % Triton X-100) and blocked (1 % BSA). Anti-S100 antibody was incubated with cells at 4 °C overnight, and the secondary was incubated with cells for 1 h. After washing with PBS, DAPI staining was performed and Images were captured.

Transcriptomic analysis was carried out to explore the underlying mechanisms of neurogenesis properties. After being co-cultured with hydrogels, cells were collected to take transcriptomic analysis. Further details are provided in Supporting Information.

### 2.6 In vitro angiogenesis properties of hydrogels

To evaluate the effect of hydrogels on angiogenesis, transwell migration assay, scratch wound assay, q-PCR, and immunofluorescent staining of HUVECs were conducted as mentioned above. The primers of angiogenesis were presented in Table S1, Supporting Information. For the tube formation assay, HUVECs were seeded on 96-well plates, which were coated with 50 μL matrigel. The cells were incubated in different hydrogel extracts for 6 h. Images were taken to measure the total tube length and number of nodes.

### 2.7 Neuroangiogenesis bioactivities to promote osteogenic differentiation in vitro

Firstly, we used a transwell migration assay to verify the recruitment properties of hydrogels with SP for BMSCs. To explore the effect of neuroangiogenesis bioactivities on BMSCs osteogenic differentiation, we constructed the conditioned medium, which was composed of HUVECs supernatant, SCs supernatant, and osteogenic induction medium at a 1:1:2 ratio, optimized based on preliminary experiments to balance neurovascular and osteogenic signals. The 1:1 ratio of HUVECs and SCs supernatants ensures equivalent contributions of angiogenic (e.g., VEGF) and neurogenic (e.g., BDNF, GDNF, NGF) factors, while the double volume of osteogenic induction medium (containing 10 nM dexamethasone, 10 mM β-glycerophosphate, and 50 μg/mL ascorbic acid) provides sufficient cues for BMSC osteogenic differentiation. This composition was aligned with prior reports on neurovascular-osteogenic crosstalk [30]. After induction with conditioned medium for 7 days and 14 days, ALP staining and ARS staining were performed. To assess the gene expression of osteogenesis, including ALP, runt-related transcription factor 2 (RUNX2), and osteocalcin (OCN), q-PCR was performed after a 7-day culture of BMSCs. The primers were presented in Table S1, Supporting Information. Immunofluorescent staining of OCN protein was also performed after a 7-day culture.

For ALP staining, BMSCs were washed with PBS 3 times and fixed with 4 % paraformaldehyde solution for 30 min. Washing with PBS 3 times again, an ALP staining working solution was used according to the manufacturer's protocol. For ARS staining, after being fixed and washed, BMSCs were incubated with an ARS staining working solution for 30 min. Washing with deionized water and images were collected.

To compare the osteogenic efficacy of the PM@PS hydrogel to an established Wnt activator, BMSCs were cultured under six conditions: Blank (DMEM with 10 % FBS and 1 % penicillin/streptomycin), Control (no hydrogel), PS (SIS-PEGDA hydrogel), P@PS (neurotrophic factor P-loaded SIS-PEGDA hydrogel), PM@PS (SP-loaded Zn-MOF integrated SIS-PEGDA hydrogel), and Wnt agonist (BML-284, 10 μM, Sigma-Aldrich). After 7 days of culture, cells were analyzed for Wnt signaling pathway activation and osteogenic differentiation via Western blot analysis. Western blot band densities were quantified using Bio-Rad Image Lab software, with GAPDH as a loading control.

### 2.8 Animal experiments

The complete protocol for animal experiments was approved by the Ethics Committee for Experimental Animals of West China Hospital, Sichuan University. In the calvarial defect model, 8-week-old male Sprague-Dawley rats were anesthetized with 2 % pentobarbital, and the skin was shaved and disinfected. A longitudinal incision was made, followed by the blunt separation of subcutaneous tissue. Then, a 6 mm, full-thickness, critical-size bone defect was created on the skull by a dental drill. The pre-hydrogel solution was injected into the defect area (Control group, treated with PBS; PS group; P@PS group; M@PS group and PM@PS group). Finally, the subcutaneous tissue was closed and the skin was sutured. Antibiotics were used to against infection. All rats were monitored daily post-operatively for general health status, wound healing, activity level, and signs of infection or discomfort. After 2, 8, and 12 weeks, the rats were euthanized and the skulls were collected and ffixed with paraformaldehyde for the follow-up studies.

Micro-computed tomography (micro-CT) was used to scan the calvarial bone. The 3D images were reconstructed via micro-CT system software. The bone mineral density (BMD) and the bone volume fraction (BV/TV) of the new bones were also analyzed. After decalcification, the calvarial bones were made into paraffin sections. Subsequently, Hematoxylin & Eosin (HE) and Masson staining were performed, respectively. The recruitment of intrinsic BMSCs was assessed via immunofluorescence staining of MSC markers (CD 44 and CD 105). S100β protein was stained to detect SCs. TUBB and CGRP staining were used to deserver the early nerve regeneration. The sections were double-stained with primary antibodies of CD31 and a-SMA to detect angiogenesis. To evaluate osteogenic differentiation, immunofluorescence staining of OCN and immunohistochemical staining of BMP-2 and Runx2 was performed.

### 2.9 Statistical analysis

The Prism software (version 9.0, GraphPad, USA) was used to draw cartograms and calculate statistical analyses via one-way analysis of variance (ANOVA) and unpaired Student's t-test. The sample size (n) of each statistical analysis has been listed in figures. *P* < 0.05 between groups was considered significant.

## 3 Results and discussion

### 3.1 Synthesis and characterization of SP@ZIF-8 nanoparticles

**Fig. 1:**
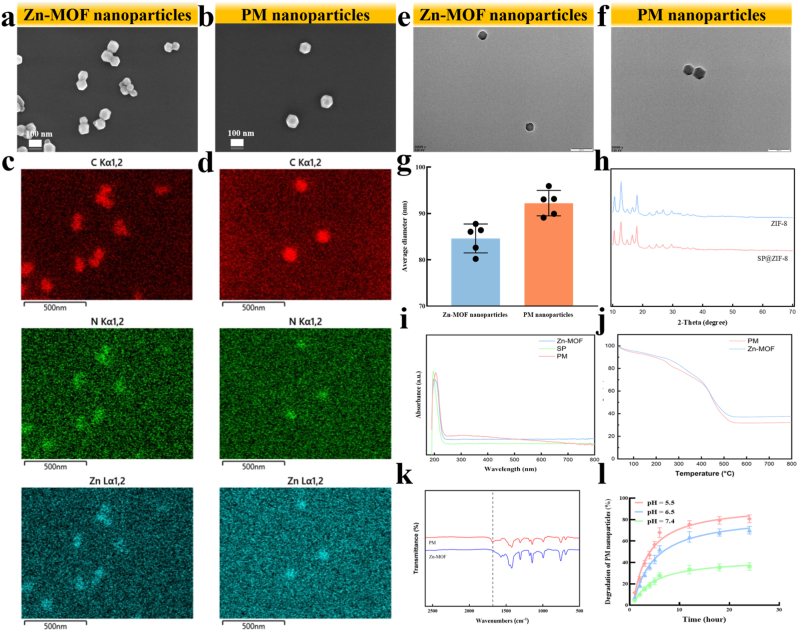
**Characterization of PM nanoparticles.** (a) SEM of Zn-MOF nanoparticles (Scale bar = 100 nm); (b) SEM of PM nanoparticles (Scale bar = 100 nm); (c) SEM-mapping of Zn-MOF nanoparticles (Scale bar = 500 nm); (d) SEM-mapping of PM nanoparticles (Scale bar = 500 nm); (e) TEM of Zn-MOF nanoparticles (Scale bar = 200 nm); (f) TEM of PM nanoparticles (Scale bar = 200 nm); (g) Average diameter of Zn-MOF and PM nanoparticles; (h) XRD of Zn-MOF and PM nanoparticles; (i) UV–vis of SP, Zn-MOF and PM nanoparticles; (j) TGA of Zn-MOF and PM nanoparticles; (k) FTIR spectrum of Zn-MOF and PM nanoparticles; (l) Degradationtendency of PM nanoparticles under different pH levels.

First, we synthesized Zn-MOF and SP-functionalized MOF (PM) nanoparticles using a hydrothermal method. SEM images revealed that the nanoparticles exhibited a typical rhombic dodecahedron shape (Fig. 1a and b). Notably, the elemental distribution in PM nanoparticles significantly changed compared to Zn-MOF due to the encapsulation of SP (Fig. 1c and d and S1). TEM was also performed to observe the typical rhombic dodecahedron morphology of Zn-MOF and PM nanoparticles (Fig. 1e and f). The average diameter of Zn-MOF nanoparticles was measured at 86.6 nm, which increased to 96.2 nm for PM nanoparticles (Fig. 1g). XRD analysis showed distinct diffraction peaks for both Zn-MOF and PM nanoparticles, consistent with previous studies, indicating that the PM nanoparticles maintained their crystalline structure (Fig. 1h).

The UV–visible spectroscopy results demonstrated a notable change between Zn-MOF and PM nanoparticles, confirming the encapsulation of SP within the nanoparticles as well (Fig. 1i). The TGA demonstrated that both Zn-MOF and PM nanoparticles exhibited weight loss, with a gradual decline between 0 and 300 °C and a more rapid loss from approximately 300 °C–500 °C. The final remaining weight percentages at temperatures from 500 °C to 800 °C were 37.7 % for Zn-MOF nanoparticles and 32.2 % for PM nanoparticles, reflecting an encapsulation efficiency of SP at 5.5 % (Fig. 1j). We also performed ELISA assays to test encapsulation efficiency, which indicated an efficiency of 5.2 %. The result closely aligns with the calculation obtained from TGA.

FTIR spectroscopy revealed similar characteristic peaks for both Zn-MOF and PM nanoparticles, with a notable shift in the peak at 1680 cm^−1^ in PM nanoparticles, indicative of SP encapsulation (Fig. 1j). Furthermore, we evaluated the degradation tendency of PM nanoparticles under different pH levels by measuring the concentration of Zn^2+^. the results demonstrated that the nanoparticles degraded more rapidly in acidic environments, indicating a pH-responsive behavior of the nanoparticles. (Fig. 1l). It has to do with the protonation effect weakens the coordination bonds between zinc ions and 2-methylimidazole [31].

### 3.2 Synthesis and characterization of the double network PM@PS hydrogel

**Fig. 2:**
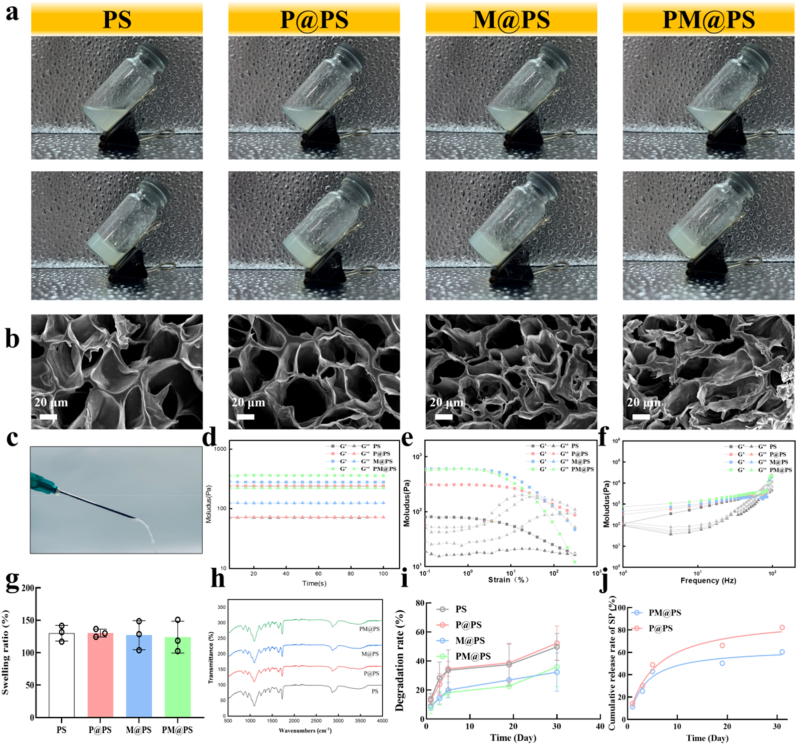
**Characterization of PM@PS hydrogel.** (a) The appearance of hydrogels before and after UV light; (b) SEM of hydrogels (Scale bar = 20 μm); (c) Injectability properties of PM@PS hydrogel; (d) The oscillatory time sweep curve of hydrogels; (e) The oscillatory strain sweep curve of hydrogels; (f) The oscillatory frequency swee sweep curve of hydrogels; (g) Swelling ratio of hydrogels; (h) The FTIR spectrums of hydrogels; (i) Degradation rate of hydrogels; (j) Cumulative release curve of SP.

Furthermore, we prepared SIS powder through enzymolysis, then added appropriate components into the PS (PEGDA/SIS) matrix to synthesize the double network hydrogels (PS, P@PS, M@PS, and PM@PS hydrogels) after double crosslinking. at room temperature (Fig. 2a). SEM imaging revealed that all hydrogels exhibited interconnected porous structures, supporting cell infiltration and vascularization. Compared to the PS hydrogel, the PM@PS hydrogel displayed more folded and undulating pore walls, potentially enhancing cell adhesion (Fig. 2b). SEM imaging revealed that all hydrogels exhibited interconnected porous structures, with the PM@PS hydrogel displaying pore sizes ranging from 100 to 180 μm, which is optimal for cell infiltration, adhesion, and vascularization, as reported in prior studies [[32], [33], [34]]. This porosity was characterized using SEM analysis of lyophilized hydrogels, a standard method in most hydrogel studies to reflect the porous architecture that influences cell behavior in the hydrated state. Compared to the PS hydrogel, the PM@PS hydrogel displayed more folded and undulating pore walls, potentially enhancing cell adhesion (Fig. 2b). The porous architecture was preserved during freeze-drying (−80 °C, 0.05 mbar) for SEM analysis, while the hydrogels are applied in their hydrated, injectable form for in vivo applications. As shown in Fig. 2c, the prepolymer solution of PM@PS was fluid and injectable, facilitating easy administration into bone defects.

To further investigate the viscoelastic properties of the hydrogels, we conducted oscillatory rheological tests. In the oscillatory time sweep, the storage modulus (G′) was consistently higher than the loss modulus (G″), indicating a stable gel structure (Fig. 2d). During the oscillatory strain sweep (0.1 %–100 %), the transition from gel to sol occurred at 72 %, suggesting that the PM@PS hydrogel could maintain its gel state under strain up to this level (Fig. 2e). The oscillatory frequency sweep demonstrated nonlinear rheological behavior, with both G′ and G″ increasing as frequency increased (Fig. 2f). In general, PM@PS and M@PS hydrogels exhibited higher G′ and G″ values than PS and P@PS hydrogels, indicating that nanoparticle incorporation enhanced the mechanical properties, making the hydrogel more suitable for bone repair.

Interestingly, PM@PS hydrogel showed a similar swelling ratio and FTIR spectrum to the other three hydrogels, indicating that the main mode of interaction is physical embedding, rather than covalent bonding, which ensures that the sustained release of SP is primarily governed by the degradation of the MOF structure and the hydrogel matrix. (Fig. 2g and h). We also examined degradation and release behaviors in vitro. PS and P@PS hydrogels degraded faster than M@PS and PM@PS, with degradation ratios of 32.3 % and 35.2 % after 30 days, suggesting that nanoparticles reduce the degradation rate (Fig. 2i). Sustained SP release is essential for long-term bone defect repair, and encapsulating SP in Zn-MOF slowed its release rate in PM@PS compared to P@PS, prolonging SP's therapeutic effects (Fig. 2j).

To assess biocompatibility, we performed a CCK-8 assay with BMSCs. On days 2 and 3, there were more cells in the PM@PS group, indicating that PM@PS hydrogels could promote BMSC growth (Fig. S2). Additionally, hemolysis testing indicated that all composite hydrogels were hemocompatible, with hemolysis ratios below 5 % after 30 min of incubation (Fig. S3).

In summary, the injectable, double-network PM@PS hydrogel demonstrated a well-structured porous architecture, improved mechanical stability, a slower degradation rate and excellent biocompatibility, which could release SP continuously, making it promising for bone tissue engineering applications.

### 3.3 Neurogenesis properties of PM@PS hydrogel in vitro

The critical role of neurogenesis in bone repair has gained increasing recognition, leading to strategies that leverage nerve regeneration to enhance bone healing [[35], [36], [37]]. SCs are pivotal in peripheral nerve repair, as they guide regenerating axons and secrete neurotrophic factors that support nerve growth [38]. To investigate the neuro-promotive effects of PM@PS hydrogel on SCs, we co-cultured rat Schwann cells (RSC 96) with the hydrogel.

**Fig. 3:**
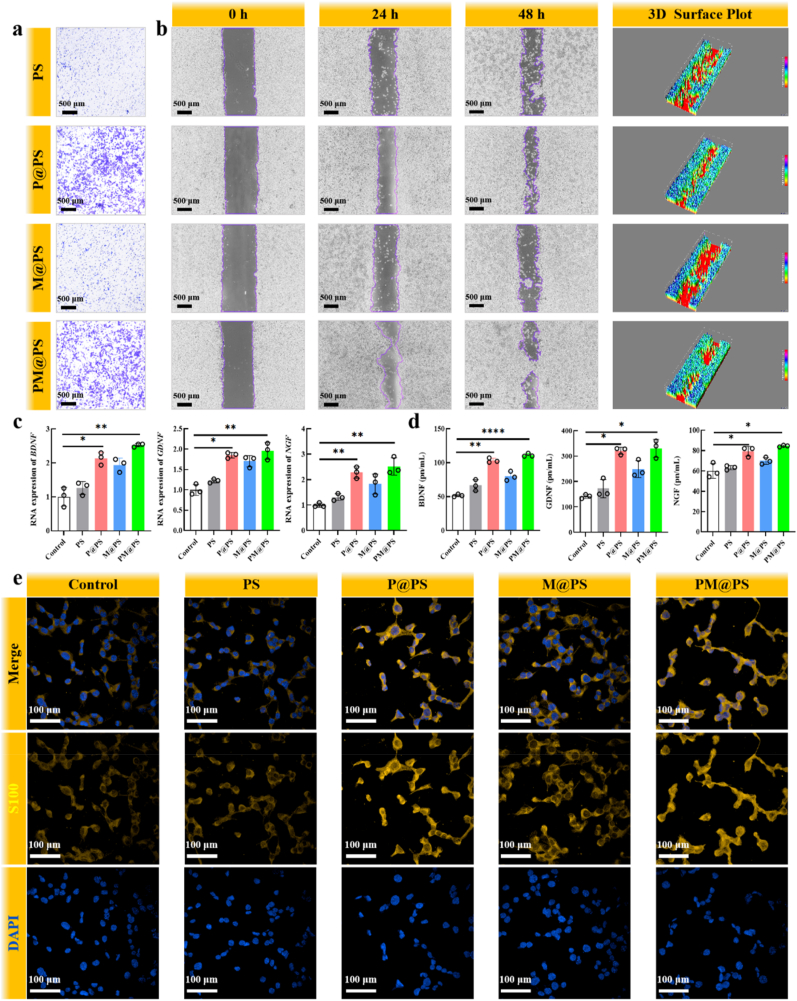
**Neurogenesis properties of PM@PS hydrogel.** (a) Transwell migration assay of SCs (Scale bar = 500 μm); (b) Scratch wound assay of SCs (Scale bar = 500 μm); (c) Genes expression levels of *BDNF*, *GDNF* and *NGF* in SCs; (d) Secretion levels of BDNF, GDNF and NGF in SCs; (e) Immunofluorescence staining of S100 (yellow) in SCs (Scale bar = 100 μm). (∗*p* < 0.05, ∗∗*p <* 0.01, ∗∗∗∗*p <* 0.0001).

Firstly, the effect of hydrogels on the proliferation of SCs was evaluated by live/dead cell staining and more living SCs were observed in the group of PM@PS hydrogel after 3-day co-cultured compared to the control group, indicating PM@PS hydrogel can promote SCs proliferation (Fig. S4). Next, we assessed the migration ability of SCs treated with different hydrogels via transwell migration assay and scratch wound assay. As shown in Fig. 3a, the number of migration cells in the P@PS and PM@PS groups is significantly more than the PS and M@PS groups, which was verified by semi-quantitative analysis (Fig. S5). In the scratch wound assay, it was clear that in the P@PS and PM@PS groups the scratch area shrinked faster than PS and M@PS groups, and the remaining scratch area in the PM@PS group was least in the 3D surface plot at 48 h (Fig. 3b). This proved that the addition of SP can observably promote SCs migration.

BDNF, GDNF, and NGF are essential neurotrophic factors for nerve growth, and SCs can synthesize and release these factors to support neuronal proliferation and regeneration [39]. To examine neurotrophic factor production, we assessed the gene expression levels of BDNF, GDNF, and NGF using qPCR. Results indicated significantly higher expression levels in the PM@PS and P@PS groups compared to controls (Fig. 3c). ELISA analysis further confirmed elevated secretion levels of these neurotrophic factors in the PM@PS and P@PS groups relative to other groups (Fig. 3d). Additionally, we immunostained S100 protein in SCs as a marker of SC activity. After two days of co-culture with the hydrogels, the P@PS and PM@PS hydrogels significantly increased S100 protein expression (Fig. 3e). Semi-quantitative fluorescence analysis further confirmed these findings (Fig. S6).

**Fig. 4:**
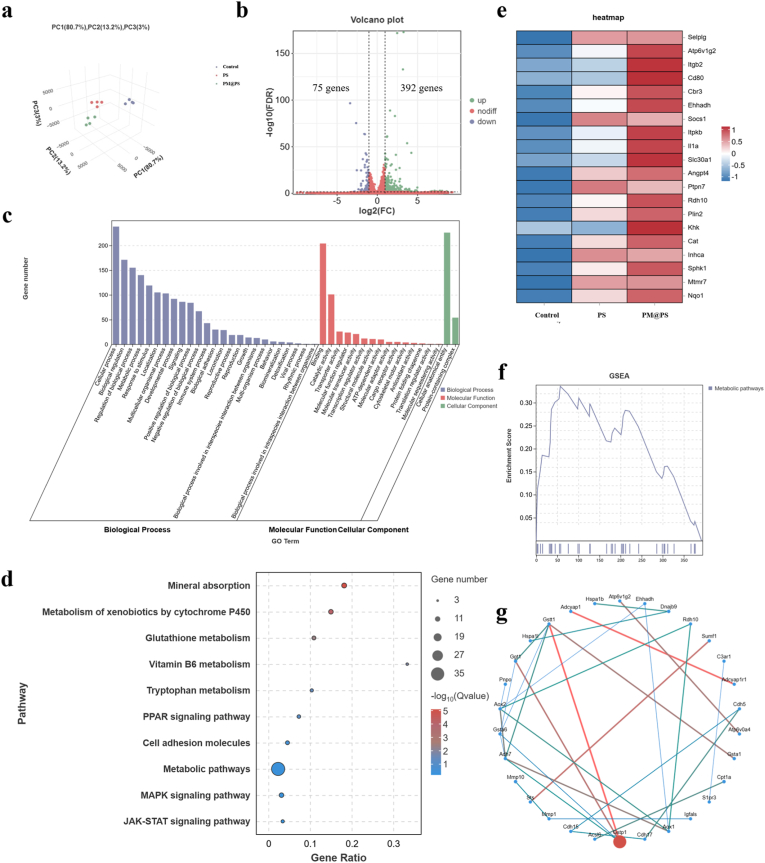
**Mechanistic analysis of PM@PS hydrogel neurogenesis effects on SCs.** (a) Principal component analysis; (b) Volcanic map; (c) Gene ontology enrichment analysis; (d) Genes and Genomes pathway enrichment analysis; (e) Heatmap analysis; (f) Gene set enrichment analysis of metabolic pathways; (g) Protein-protein interaction gene network analysis.

To investigate the mechanism by which the PM@PS hydrogel promotes neurogenesis, we conducted mRNA sequencing of SCs after co-culturing with PS and PM@PS hydrogels for two days. Principal Component Analysis (PCA) confirmed satisfactory within-group reproducibility and clear inter-group discrimination of the samples (Fig. 4a). A volcano plot revealed 392 up-regulated and 75 down-regulated differentially expressed genes in the PM@PS group compared to the control group (Fig. 4b).

Subsequently, we performed Gene Ontology (GO) enrichment analysis and Kyoto Encyclopedia of Genes and Genomes (KEGG) pathway analysis on the up-regulated genes. The GO analysis indicated that the PM@PS hydrogel influences SC functions by modulating biological processes (BP), including cellular processes, biological regulation, metabolic processes, biological adhesion, signaling, growth, locomotion, responses to stimuli, and biomineralization. Molecular functions (MF) affected included cytoskeletal motor activity, catalytic activity, structural molecule activity, transporter activity, binding, antioxidant activity, cargo receptor activity, protein folding chaperone activity, translation regulator activity, molecular transducer activity, molecular function regulation, transcription regulation activity, and molecular sequestering activity. The cellular components (CC) involved included protein-containing complexes and cellular anatomical entities (Fig. 4c). KEGG analysis highlighted significant signaling pathways related to various metabolic processes, such as glutathione metabolism, vitamin B6 metabolism, and tryptophan metabolism. The results also identified significant enrichment of PPAR, MAPK, and JAK-STAT signaling pathways, which are related to nerve repair. And the PPAR pathway showing the most prominent activation (Fig. 4d). Notably, the PPAR signaling pathway is known for its neuroprotective roles in various neurological disorders and its contribution to pro-regenerative responses following nerve injury [40,41]. The MAPK signaling pathway mediates cell growth and axon regeneration, while the JAK-STAT pathway is implicated in regulating SCs migration [[42], [43], [44]]. To validate the PPAR pathway, we performed qRT-PCR for *PPARa, RXRA,* and *LPL*, confirming significant upregulation in the PM@PS group (Fig. S7). Hierarchical clustering analysis generated a heat map, revealing that the genes associated with these signaling pathways were significantly upregulated in the PM@PS group compared to the control group (Fig. 4e). Gene Set Enrichment Analysis (GSEA) further confirmed the activation of metabolic pathways in the PM@PS group (Fig. 4f). Additionally, protein-protein interaction (PPI) network analysis indicated that the gene Gstp1 exhibited the highest abundance among the upregulated genes, which has been previously associated with the regulation of neuritogenesis (Fig. 4g) [45].

To investigate the neurogenic effects of the PM@PS hydrogel, we assessed Schwann cell (SC) behavior through immunofluorescence staining for S100 protein, which showed enhanced expression in the PM@PS group, indicating promoted SC differentiation (Fig. 3). As a supplementary analysis, we conducted mRNA sequencing of SCs after co-culturing with PS and PM@PS hydrogels for two days. KEGG analysis highlighted significant signaling pathways related to nerve repair, including the PPAR, MAPK, and JAK-STAT signaling pathways (Fig. 4d). These transcriptomic data, combined with the immunofluorescence results and neurogenic marker data in Fig. 3, provide robust evidence of the hydrogel's neurogenic effects.

In summary, the PM@PS hydrogel positively influences neurogenesis by enhancing SC proliferation, migration, and neurotrophic factor secretion, supporting its potential to facilitate myelination and promote neural repair. The underlying mechanism may involve the activation of multiple nerve regeneration-related signaling pathways and GSTP1 may play an important role in this process.

### 3.4 Angiogenesis properties of PM@PS hydrogel in vitro

**Fig. 5:**
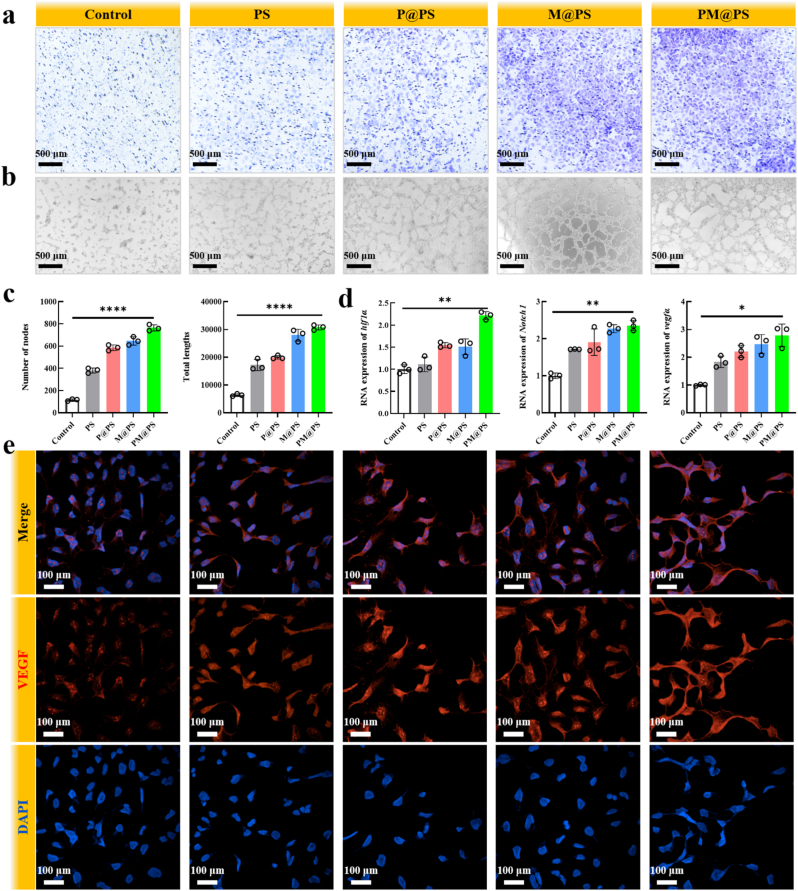
**Angiogenesis properties of PM@PS hydrogel.** (a) Transwell migration assay of HUVECs (Scale bar = 500 μm); (b) Tube formation assay of HUVECs (Scale bar = 500 μm); (c) Number of nodes and tube total length; (d) Genes expression levels of *hif1α, Notch1* and *vegfα* in HUEVCs; (e) Immunofluorescence staining of VEGF (red) in HUEVCs (Scale bar = 100 μm). (∗*p* < 0.05, ∗∗*p <* 0.01, ∗∗∗∗*p <* 0.0001).

Angiogenesis is also important for bone regeneration, as the vascular network within bone tissue ensures the delivery of oxygen, essential nutrients, mineralized components, and growth factors necessary for osteogenesis [46,47]. We conducted assays to evaluate the angiogenic properties of the PM@PS hydrogel in relation to HUVECs. First, we assessed the migratory capacity of HUVECs using transwell migration and scratch wound assays. As shown in Fig. 5a, the number of migrating HUVECs increased across all hydrogel groups compared to the control group, with the M@PS and PM@PS groups exhibiting the highest migration rates. This enhanced migration was attributed to SP and the Zn^2+^ ions released from Zn-MOF, which are known to modulate cellular signaling and promote cell migration [48]. Further semi-quantitative analysis confirmed that the PM@PS group had the highest number of migrating cells (Fig. S8), and similar trends were observed in the scratch wound assay (Fig. S9).

The in *vitro* formation of capillary-like tubes by endothelial cells on a basement membrane matrix is a powerful method to assess angiogenesis properties [49]. To evaluate the formation of capillary-like structures, we cultured HUVECs on a basement membrane matrix for 6 h. Vessel-like networks were observed in the PS, P@PS, M@PS, and PM@PS groups (Fig. 5b). Image analysis of key parameters, including the number of nodes and total tube length, revealed that the PM@PS hydrogel group exhibited the highest number of nodes and the longest tube lengths (Fig. 5c).

We also examined the expression of angiogenesis-related genes, including *HIF1α*, *Notch1*, *and VEGFα*. QPCR results of the gene expression in all groups with hydrogels showed a rise of varying degrees and the PM@PS group had the highest improvement, indicating that the PM@PS hydrogel could significantly enhance angiogenic activity (Fig. 5d). Additionally, immunofluorescent staining for VEGF revealed positive staining in all hydrogel groups (Fig. 5e). Semi-quantitative analysis of fluorescence intensity showed that the PM@PS group exhibited the highest intensity, further supporting its angiogenic potential (Fig. S10).

In summary, our findings demonstrate that the PM@PS hydrogel can effectively accelerate HUVEC migration, facilitate the formation of capillary-like structures, and enhance the expression of angiogenesis-related genes and proteins.

### 3.5 Neuroangiogenesis bioactivities of PM@PS hydrogel to promote osteogenic differentiation in vitro

**Fig. 6:**
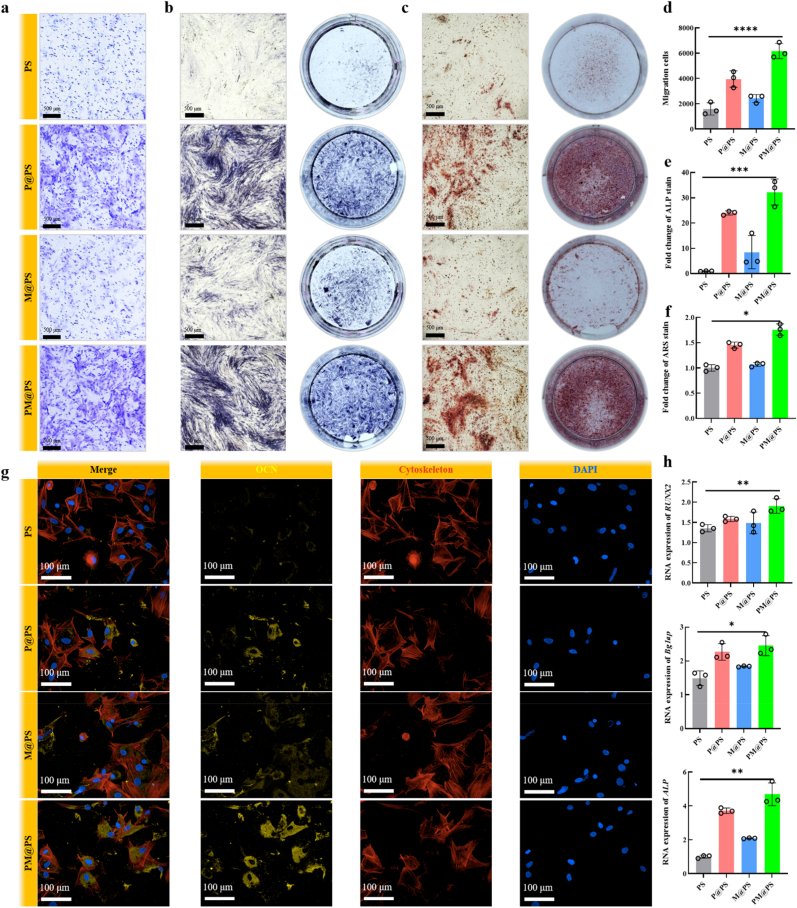
**Neurogenesis bioactivities of PM@PS hydrogel to promote osteogenic differentiation.** (a) Transwell migration assay of BMSCs (Scale bar = 500 μm); (b) ALP staining (Scale bar = 500 μm); (c) ARS staining (Scale bar = 500 μm); (d) The number of migrating cells; (e) semi-quantitative assays of ALP staining; (f) semi-quantitative assays of ARS staining; (g) Immunofluorescence staining of OCN (yellow) and cytoskeleton (red) in BMSCs (Scale bar = 100 μm); (h) Genes expression levels of *RUNX2, Bglap and ALP* in BMSCs. (∗*p* < 0.05, ∗∗*p <* 0.01, ∗∗∗*p <* 0.001, ∗∗∗∗*p <* 0.0001).

To evaluate the effects of the PM@PS hydrogel on BMSCs, we extracted BMSCs from rats and cultured with hydrogels. Initially, we assessed BMSC proliferation through live/dead cell staining. The results indicated that all hydrogels displayed excellent biocompatibility with BMSCs and the PM@PS group exhibited a higher cell density compared to other groups after 3 days of culture (Fig. S11), suggesting that the PM@PS hydrogel has excellent cell compatibility and can promote BMSC proliferation. Early recruitment of stem cells is essential for effective bone regeneration. To assess the BMSC recruitment potential of the PM@PS hydrogel, we performed a transwell migration assay. As shown in Fig. 6a, a significant number of migrating BMSCs were observed in the P@PS and PM@PS groups. Quantitative analysis confirmed that the PM@PS hydrogel was the most effective at promoting stem cell migration (Fig. 4d).

To further evaluate the osteogenic potential from neuroangiogenesis bioactivities of hydrogels, we investigated the osteogenic differentiation of BMSCs treated with conditional medium, consisting of HUVECS supernatant, SCs supernatant and osteogenic induction medium. We performed ALP staining at day 7 and ARS staining at day 14. The ALP staining intensity was significantly greater in the P@PS and PM@PS groups compared to the PS and M@PS groups (Fig. 6b), with the PM@PS group showing the strongest staining intensity in semi-quantitative assays (Fig. 6e). The ARS staining results found abundant mineralized nodules in the SP-containing groups after 14 days, while few nodules were observed in the PS and M@PS groups (Fig. 6c and f). Next, we evaluated the expression of osteogenic differentiation genes, including *RUNX2*, *Bglap*, and *ALP* (Fig. 6h). The results showed that the expression of these osteogenic-related genes was significantly higher in the PM@PS group compared to other groups. Immunofluorescence staining revealed that the P@PS and PM@PS groups significantly upregulated the synthesis of OCN protein (Fig. 6g), with the PM@PS group exhibiting the highest fluorescence intensity in semi-quantitative analysis than other groups (Fig. S12). These results suggest that the conditioned medium derived from the PM@PS group exerts a strong osteogenic effect on BMSCs, indicating that the PM@PS hydrogel can further regulate BMSC osteogenic differentiation by promoting neuro-vascular regeneration.

**Fig. 7:**
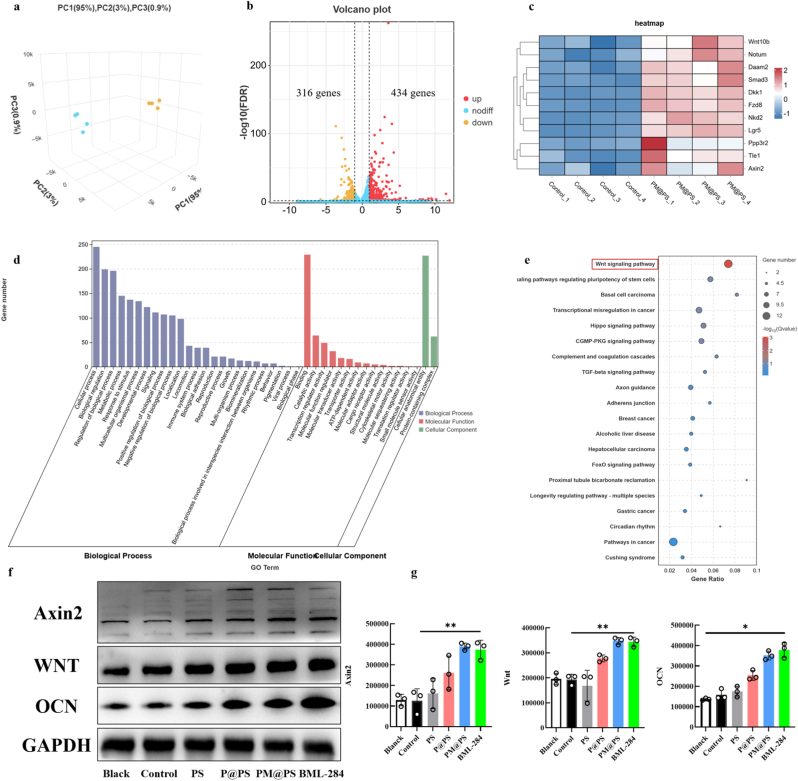
**Mechanistic analysis of neurogenesis bioactivities promotes osteogenesis.** (a) Principal component analysis; (b) Volcanic map; (c) Heatmap analysis; (d) Gene ontology enrichment analysis; (e) Genes and Genomes pathway enrichment analysis; (f) Bands of Wnt signaling pathway-related proteins and OCN protein; (g) Quantification of proteins expression. (∗*p* < 0.05, ∗∗*p <* 0.01).

To gain deeper insights into the osteogenic capacities, we conducted mRNA sequencing and compared the conditional medium from the Control group with that from the PM@PS group. PCA revealed distinct clustering, with the PM@PS group clearly separated from the Control group, indicating stability in the components and significant differences between the groups (Fig. 7a). The analysis of differentially expressed genes identified 434 up-regulated and 316 down-regulated genes, as illustrated in the volcano plot (Fig. 7b). Further analysis of the up-regulated genes through GO enrichment revealed significant BP, MF, and CC (Fig. 7d). KEGG analysis indicated that the Wnt signaling pathway was significantly activated, which is known to play a crucial role in osteoblast differentiation and bone formation. Additional pathways associated with bone regeneration and BMSCs were also identified, including those regulating the pluripotency of stem cells, the Hippo signaling pathway, the cGMP-PKG signaling pathway, and the TGF-beta signaling pathway (Fig. 7e). A heatmap illustrating the differentially expressed genes associated with the Wnt signaling pathways is shown in Fig. 7c. To validate the results of the transcriptome, we compared its effects on BMSCs to BML-284 (a known Wnt agonist) and detected the expression of Wnt signaling pathway-related proteins and OCN protein (Fig. 7f). The semi-quantitative results revealed significant activation of the Wnt signaling pathway in both the PM@PS and BML-284 groups, with increased expression of Wnt signaling pathway-related proteins and OCN compared to the other groups. Original uncut images used for quantitative analysis are provided in Fig. S13. These findings suggest that the PM@PS hydrogel effectively promotes osteogenic differentiation, likely mediated by enhanced neurovascular activity and activation of the Wnt signaling pathway. Nevertheless, further studies are needed to elucidate the underlying mechanisms of cell–material interactions and intercellular signaling crosstalk.

### 3.6 p.m.@PS hydrogel promotes bone repair in vivo calvarial bone defect model

Based on the results described above, the PM@PS hydrogel exhibited significant neurogenic, angiogenic, and osteogenic effects, demonstrating strong potential for promoting bone defect repair. To further evaluate its bioactivity in vivo, we established a 6 mm critical-size calvarial defect model in Sprague–Dawley rats. The pre-gel solution was injected into the defect area and crosslinked in situ. Skulls were harvested at multiple time points for further analysis.

**Fig. 8:**
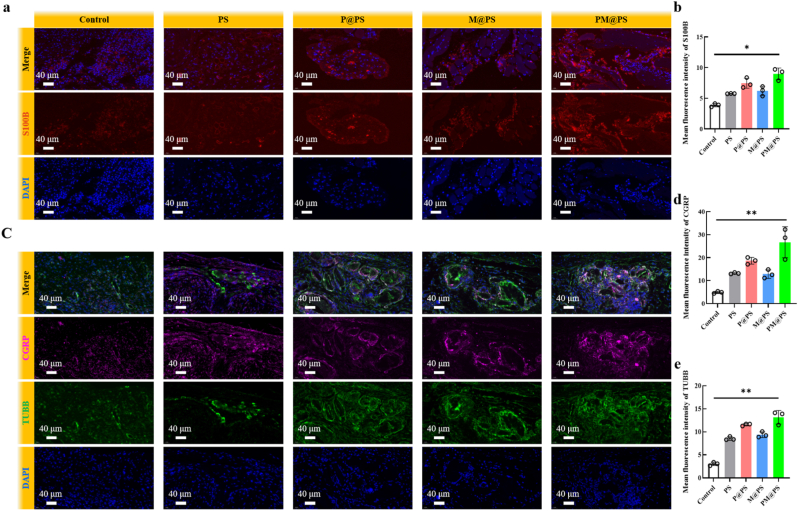
**The PM@PS hydrogel enhanced neurogenesis in vivo.** (a) Immunofluorescence staining of S100B (red) in the defect zones at 2 weeks (Scale bar = 40 μm); (b) Semi-quantification of S100B; (c) Immunofluorescence staining of CGRP (purple) and TUBB (green) in the defect zones at 2 weeks (Scale bar = 40 μm); (d) Semi-quantification of CGRP; (e) Semi-quantification of TUBB. (∗*p* < 0.05, ∗∗*p <* 0.01).

Innervation plays a critical role in the early stages of bone regeneration, with Schwann cells (SCs) contributing to remyelination and the secretion of neurotrophic factors. To evaluate SC recruitment, immunofluorescence staining for S100B, a Schwann cell marker, was performed at 2 weeks post-surgery. As shown in Fig. 8a, significantly larger S100B-positive areas were observed in the P@PS and PM@PS groups compared to the Control group. Semi-quantitative analysis further confirmed that the PM@PS hydrogel markedly promoted SC recruitment and proliferation in the defect region (Fig. 8b). Given that CGRP-positive nerve fibers are prevalent in bone tissue [50], we further examined the distribution of regenerating nerves by co-staining for CGRP and TUBB3. The P@PS, M@PS, and PM@PS groups all showed higher levels of CGRP/TUBB3-positive fibers compared to the Control group, with the PM@PS group exhibiting the most abundant nerve fiber ingrowth (Fig. 8c–e). These findings demonstrate that the PM@PS hydrogel effectively facilitates nerve infiltration into the defect site.

**Fig. 9:**
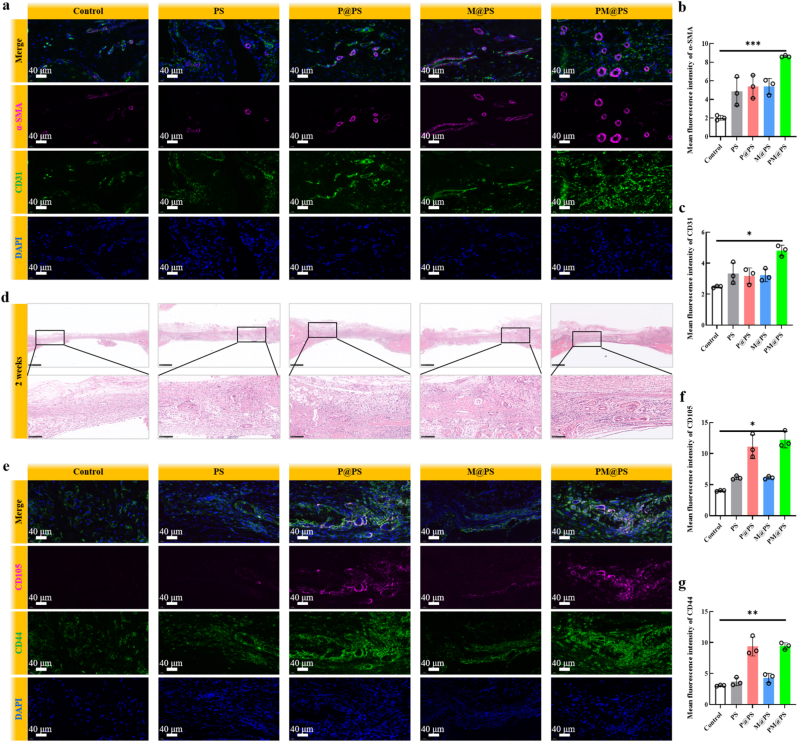
**The PM@PS hydrogel enhanced angiogenesis and BMSCs recruitment in vivo.** (a) Immunofluorescence staining of α-SMA (purple) and CD31 (green) in the defect zones at 2 weeks (Scale bar = 40 μm); (b) Semi-quantification of α-SMA; (c) Semi-quantification of CD31; (d) HE staining of the critical size defect zones at 2 weeks (Scale bar = 500 μm in the entire defect, scale bar = 100 μm in the local enlargement); (e) Immunofluorescence staining of CD105 (purple) and CD44 (green) in the defect zones at 2 weeks (Scale bar = 40 μm); (f) Semi-quantification of CD105; (g) Semi-quantification of CD44. (∗*p* < 0.05, ∗∗*p <* 0.01, ∗∗∗*p <* 0.001).

Angiogenesis is another key component of successful bone healing [51]. To assess neovascularization, we performed CD31 and α-SMA immunofluorescence staining in the regenerated area. The P@PS and PM@PS groups exhibited substantially more vessel-like structures co-expressing CD31 and α-SMA than the Control, PS, or M@PS groups. While some non-specific staining may contribute to the high fluorescence intensity in the PM@PS group, more intense green CD31 staining is observed surrounding tubular vascular structures, confirming specific endothelial cell labeling. Semi-quantification showed that the highest fluorescence intensity observed in the PM@PS group (Fig. 9b and c).

In addition, H&E staining at 2 weeks revealed a greater density of newly formed blood vessels in the PM@PS group (Fig. 9d), further supporting the hydrogel's pro-angiogenic potential. It is important to note that the in vivo angiogenesis effects observed here may differ from the in vitro results due to the complexity of the in vivo environment in the M@PS and P@PS groups. In vivo, the neuropeptide SP in the PM@PS hydrogel likely plays a more pronounced role in promoting angiogenesis due to the intricate interactions within the tissue, including immune responses and the involvement of various cellular components and growth factors. These factors are not present in the simplified in vitro system, which may explain the observed differences in angiogenesis potential between the experimental setups [29,52]. Despite these variations, both in vitro and in vivo experiments demonstrated the most prominent angiogenesis effects in the PM@PS group.

To investigate the recruitment of MSCs, we conducted immunofluorescence staining for CD44 and CD105 at 2 weeks post-surgery. The P@PS and PM@PS groups showed significantly more CD44^+^/CD105^+^ cells within the defect area, indicating enhanced early MSC recruitment by SP-containing hydrogels (Fig. 9e–g). This early recruitment of progenitor cells is crucial for subsequent osteogenesis.

**Fig. 10:**
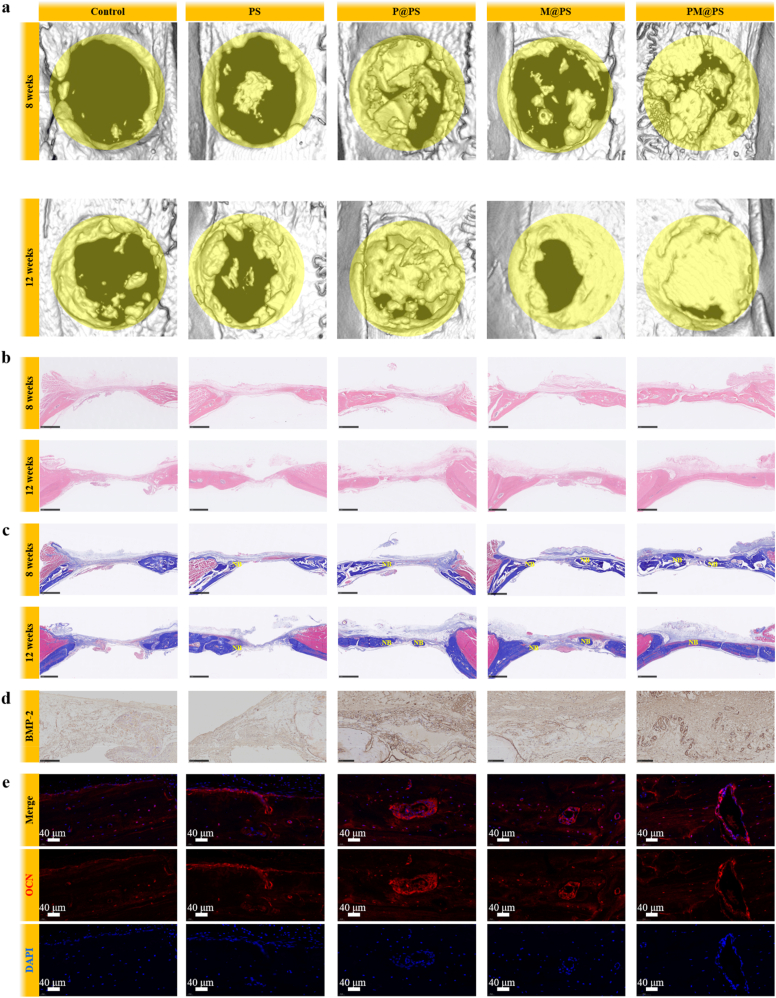
**The PM@PS hydrogel enhanced osteogenesis in vivo.** (a) Representative images of micro-CT at 8 weeks and 12 weeks. Regenerated bone areas are outlined in yellow to indicate the defect boundary; (b) HE staining of the critical size defect zones at 8 and 12 weeks (Scale bar = 500 μm), NB: newly formed bone tissue; (c) Masson staining of the critical size defect zones at 8 and 12 weeks (Scale bar = 500 μm); (d) Immunohistochemical staining of BMP-2 in the defect zones at 12 weeks (Scale bar = 250 μm); (e) Immunofluorescence staining of OCN (red) in the defect zones at 12 weeks (Scale bar = 40 μm).

To evaluate bone formation, micro-CT scans at 8 weeks and 12 weeks post-implantation revealed robust new bone growth in the P@PS and PM@PS groups. By 12 weeks, the PM@PS group showed nearly complete defect closure, whereas the Control group still presented with visible cavities and limited peripheral bone formation. The PS and M@PS groups exhibited moderate repair (Fig. 10a). BV/TV analysis confirmed that the PM@PS group had significantly higher bone volume relative to total volume compared to all other groups (Fig. S14).

H&E and Masson staining showed a similar pattern: minimal new bone and collagen in the Control group, but substantial collagen fiber formation and regenerated bone tissue in the PM@PS group (Fig. 10b and c). Further confirmation of osteogenesis was obtained through immunohistochemical staining for BMP-2 and RUNX2, two key osteogenic markers at 12 weeks. Their expression was markedly increased in the P@PS and PM@PS groups, with the PM@PS group showing the highest levels (Fig. 10d and Fig. S15). OCN immunofluorescence staining revealed strong red fluorescence in the PM@PS group, while only weak signals were detected in the Control group at 12 weeks (Fig. 10e). Quantitative analysis of fluorescence intensity further supported these observations (Fig. S16), indicating that PM@PS significantly promoted mineralization and bone matrix maturation. To provide a comprehensive view of bone regeneration, we supplemented 8-week staining data for BMP-2, RUNX2, and OCN, which show early bone formation, with significantly higher expression in the PM@PS group (Fig. S17).

Lastly, to confirm biosafety, we performed routine blood tests and serum biochemical analyses. The results indicated that both liver and kidney functions remained within normal ranges in rats treated with the hydrogel, suggesting that the PM@PS system is safe for in vivo applications (Fig. S18).

Collectively, the in vivo results demonstrate that the PM@PS hydrogel promotes coordinated neurogenesis, angiogenesis, and osteogenesis within the bone defect microenvironment. Early recruitment of Schwann cells, vascular endothelial cells, and BMSCs—coupled with enhanced neurovascular network formation—contributed to a favorable regenerative niche, which significantly accelerated new bone formation and mineralization. Previous studies have suggested that when peripheral nerves are injured at the fracture site, damage signals are retrogradely transmitted along the proximal axons to the cell body, triggering the regeneration process [53]. Neuropeptides such as CGRP and SP, which are released during the early stage of acute injuries, can stimulate Schwann cells and endothelial cells, enhancing neurogenesis and angiogenesis, respectively. This further creates a supportive microenvironment for osteogenesis. Neurogenesis can regulate osteogenesis through neurotrophic factors like BDNF, GDNF, and NGF. Angiogenesis ensures the delivery of oxygen, nutrients, and growth factors necessary for osteoblast differentiation and bone matrix formation. By exogenously supplementing the neuropeptide SP, we simulate the neuropeptides released during the acute phase of injury regeneration. This enhances neurogenesis and angiogenesis. Together, they create a favorable environment with a neurovascular network for bone regeneration.

## 4 Conclusion

In summary, we successfully developed an advanced neurovascular-driven bone repair system for bone defects by combining ECM materials with SP-loaded nano MOF to create a efficient, safe and low-cost repair hydrogel. This injectable and double network hydrogel facilitates easy implantation and precise adaptation to bone defects while forming a stable, supportive matrix. Both in vitro and in vivo studies demonstrated that the hydrogel exhibits excellent biocompatibility. The ECM components derived from SIS impart strong angiogenic properties to the hydrogel. During degradation, the PM@PS hydrogel gradually releases SP and Zn^2+^, effectively recruiting SCs and BMSCs to the defect site. This system promotes nerve regeneration by activating the PPAR, MAPK, and JAK-STAT signaling pathways. Additionally, the hydrogel enhances neuro-vascular network formation and facilitates subsequent osteogenic differentiation of BMSCs through upregulation of the Wnt signaling pathway. These findings underscore the therapeutic potential of neurovascular-driven bone regeneration strategies, positioning this system as a promising solution for bone tissue repair and regeneration in future biomedical applications.

## CRediT authorship contribution statement

**Ning Sheng:** Writing – original draft. **Runze Yang:** Writing – original draft. **Jie Wang:** Writing – original draft. **Wenting Wu:** Methodology. **Man Zhe:** Methodology. **Qing-Yi Zhang:** Methodology. **Rong Nie:** Methodology. **Long Chen:** Methodology. **Fei Xing:** Writing – review & editing. **Li Sun:** Writing – review & editing.

## Declaration of competing interest

This manuscript has been approved by all authors. It has not been published or presented elsewhere, in whole or in part, and is not under consideration by another journal. We have read and understood the policies of your journal and believe that neither the manuscript nor the study violates any of them. There are no conflicts of interest to declare.

## Data Availability

Data will be made available on request.

## References

[1] You J., Li Y., Wang C., Lv H., Zhai S., Liu M., Liu X., Sezhen Q., Zhang L., Zhang Y., Zhou Y. (2024). Mild thermotherapy-assisted GelMA/HA/MPDA@Roxadustat 3D-Printed scaffolds with combined angiogenesis-osteogenesis functions for bone regeneration. Adv Healthc Mater.

[2] Wang Y., Li W., Guo Y., Huang Y., Guo Y., Song J., Mei F., Liao P., Gong Z., Chi X., Deng X. (2024). Mitochondria transplantation to bone marrow stromal cells promotes angiogenesis during bone repair. Adv. Sci. (Weinh.).

[3] Zhang Z., Hao Z., Xian C., Fang Y., Cheng B., Wu J., Xia J. (2022). Neuro-bone tissue engineering: multiple potential translational strategies between nerve and bone. Acta Biomater..

[4] Wan Q.Q., Qin W.P., Ma Y.X., Shen M.J., Li J., Zhang Z.B., Chen J.H., Tay F.R., Niu L.N., Jiao K. (2021). Crosstalk between bone and nerves within bone. Adv. Sci. (Weinh.).

[5] Li Z., Meyers C.A., Chang L., Lee S., Li Z., Tomlinson R., Hoke A., Clemens T.L., James A.W. (2019). Fracture repair requires TrkA signaling by skeletal sensory nerves. J. Clin. Investig..

[6] Zhou R., Yuan Z., Liu J., Liu J. (2016). Calcitonin gene-related peptide promotes the expression of osteoblastic genes and activates the WNT signal transduction pathway in bone marrow stromal stem cells. Mol. Med. Rep..

[7] Jia S., Zhang S.J., Wang X.D., Yang Z.H., Sun Y.N., Gupta A., Hou R., Lei D.L., Hu K.J., Ye W.M., Wang L. (2019). Calcitonin gene-related peptide enhances osteogenic differentiation and recruitment of bone marrow mesenchymal stem cells in rats. Exp. Ther. Med..

[8] Shi L., Wang C., Yan Y., Wang G., Zhang J., Feng L., Yang X., Li G. (2020). Function study of vasoactive intestinal peptide on chick embryonic bone development. Neuropeptides.

[9] Zhang W., Lyu M., Bessman N.J., Xie Z., Arifuzzaman M., Yano H., Parkhurst C.N., Chu C., Zhou L., Putzel G.G., Li T.T., Jin W.B., Zhou J., Hu H., Tsou A.M., Guo C.J., Artis D. (2022). Gut-innervating nociceptors regulate the intestinal microbiota to promote tissue protection. Cell.

[10] Redkiewicz P. (2022). The regenerative potential of substance P. Int. J. Mol. Sci..

[11] Park D., Kim D., Park S.J., Choi J.H., Seo Y., Kim D.H., Lee S.H., Hyun J.K., Yoo J., Jung Y., Kim S.H. (2022). Micropattern-based nerve guidance conduit with hundreds of microchannels and stem cell recruitment for nerve regeneration. NPJ Regen. Med..

[12] Kim D., Park D., Kim T.H., Chung J.J., Jung Y., Kim S.H. (2021). Substance P/Heparin-Conjugated PLCL mitigate acute gliosis on neural implants and improve neuronal regeneration via recruitment of neural stem cells. Adv Healthc Mater.

[13] Kim S.J., Kim J.E., Kim S.H., Kim S.J., Jeon S.J., Kim S.H., Jung Y. (2016). Therapeutic effects of neuropeptide substance P coupled with self-assembled peptide nanofibers on the progression of osteoarthritis in a rat model. Biomaterials.

[14] Wang Y., Zeng M., Fan T., Jia M., Yin R., Xue J., Xian L., Fan P., Zhan M. (2024). Biomimetic ZIF-8 nanoparticles: a novel approach for biomimetic drug delivery systems. Int J Nanomedicine.

[15] Gong J., Ye C., Ran J., Xiong X., Fang X., Zhou X., Yi Y., Lu X., Wang J., Xie C., Liu J. (2023). Polydopamine-mediated immunomodulatory patch for diabetic periodontal tissue regeneration assisted by Metformin-ZIF system. ACS Nano.

[16] Kang Y., Xu C., Meng L., Dong X., Qi M., Jiang D. (2022). Exosome-functionalized magnesium-organic framework-based scaffolds with osteogenic, angiogenic and anti-inflammatory properties for accelerated bone regeneration. Bioact. Mater..

[17] Tan J., Li S., Sun C., Bao G., Liu M., Jing Z., Fu H., Sun Y., Yang Q., Zheng Y., Wang X., Yang H. (2024). A dose-dependent spatiotemporal response of angiogenesis elicited by Zn biodegradation during the initial stage of bone regeneration. Adv Healthc Mater.

[18] Dhivya S., Ajita J., Selvamurugan N. (2015). Metallic nanomaterials for bone tissue engineering. J. Biomed. Nanotechnol..

[19] Zhou H., Jing S., Xiong W., Zhu Y., Duan X., Li R., Peng Y., Kumeria T., He Y., Ye Q. (2023). Metal-organic framework materials promote neural differentiation of dental pulp stem cells in spinal cord injury. J Nanobiotechnology.

[20] Liu Y., Li T., Sun M., Cheng Z., Jia W., Jiao K., Wang S., Jiang K., Yang Y., Dai Z., Liu L., Liu G., Luo Y. (2022). ZIF-8 modified multifunctional injectable photopolymerizable GelMA hydrogel for the treatment of periodontitis. Acta Biomater..

[21] Li D., Chen K., Tang H., Hu S., Xin L., Jing X., He Q., Wang S., Song J., Mei L., Cannon R.D., Ji P., Wang H., Chen T. (2022). A logic-based diagnostic and therapeutic hydrogel with multistimuli responsiveness to orchestrate diabetic bone regeneration. Adv Mater.

[22] Wang W., Zhang G., Wang Y., Ran J., Chen L., Wei Z., Zou H., Cai Y., Han W. (2023). An injectable and thermosensitive hydrogel with nano-aided NIR-II phototherapeutic and chemical effects for periodontal antibacteria and bone regeneration. J Nanobiotechnology.

[23] Nie R., Zhang Q.-Y., Tan J., Feng Z.-Y., Huang K., Sheng N., Jiang Y.-L., Song Y.-T., Zou C.-Y., Zhao L.-M., Li H.-X., Wang R., Zhou X.-L., Hu J.-J., Wu C.-Y., Li-Ling J., Xie H.-Q. (2023). EGCG modified small intestine submucosa promotes wound healing through immunomodulation. Compos. B Eng..

[24] Brown M., Li J., Moraes C., Tabrizian M., Li-Jessen N.Y.K. (2022). Decellularized extracellular matrix: new promising and challenging biomaterials for regenerative medicine. Biomaterials.

[25] Choi J.W., Park J.K., Chang J.W., Kim D.Y., Kim M.S., Shin Y.S., Kim C.H. (2014). Small intestine submucosa and mesenchymal stem cells composite gel for scarless vocal fold regeneration. Biomaterials.

[26] Sheng N., Xing F., Zhang Q.-Y., Tan J., Nie R., Huang K., Li H.-X., Jiang Y.-L., Tan B., Xiang Z., Xie H.-Q. (2024). A pleiotropic SIS-based hydrogel with immunomodulation via NLRP3 inflammasome inhibition for diabetic bone regeneration. Chem. Eng. J..

[27] Tan J., Zhang Q.-Y., Song Y.-T., Huang K., Jiang Y.-L., Chen J., Wang R., Zou C.-Y., Li Q.-J., Qin B.-Q., Sheng N., Nie R., Feng Z.-Y., Yang D.-Z., Yi W.-H., Xie H.-Q. (2022). Accelerated bone defect regeneration through sequential activation of the M1 and M2 phenotypes of macrophages by a composite BMP-2@SIS hydrogel: an immunomodulatory perspective. Compos. B Eng..

[28] Chen T.T., Yi J.T., Zhao Y.Y., Chu X. (2018). Biomineralized metal-organic framework nanoparticles enable intracellular delivery and endo-lysosomal release of native active proteins. J. Am. Chem. Soc..

[29] Hong H.S., Lee J., Lee E., Kwon Y.S., Lee E., Ahn W., Jiang M.H., Kim J.C., Son Y. (2009). A new role of substance P as an injury-inducible messenger for mobilization of CD29(+) stromal-like cells. Nat Med.

[30] Xia Y., Jing X., Wu X., Zhuang P., Guo X., Dai H. (2023). 3D-printed dual-ion chronological release functional platform reconstructs neuro-vascularization network for critical-sized bone defect regeneration. Chem. Eng. J..

[31] Kai M., Wang S., Gao W., Zhang L. (2023). Designs of metal-organic framework nanoparticles for protein delivery. J Control Release.

[32] Tang D., Tare R.S., Yang L.-Y., Williams D.F., Ou K.-L., Oreffo R.O.C. (2016). Biofabrication of bone tissue: approaches, challenges and translation for bone regeneration. Biomaterials.

[33] Murphy C.M., Haugh M.G., O'Brien F.J. (2010). The effect of mean pore size on cell attachment, proliferation and migration in collagen–glycosaminoglycan scaffolds for bone tissue engineering. Biomaterials.

[34] Abbasi N., Hamlet S., Love R.M., Nguyen N.-T. (2020). Porous scaffolds for bone regeneration. J. Sci. Adv. Mater. Devices.

[35] Xu Y., Xu C., Yang K., Ma L., Li G., Shi Y., Feng X., Tan L., Duan D., Luo Z., Yang C. (2023). Copper ion-modified germanium phosphorus nanosheets integrated with an electroactive and biodegradable hydrogel for neuro-vascularized bone regeneration. Adv Healthc Mater.

[36] Lian M., Qiao Z., Qiao S., Zhang X., Lin J., Xu R., Zhu N., Tang T., Huang Z., Jiang W., Shi J., Hao Y., Lai H., Dai K. (2024). Nerve growth factor-preconditioned mesenchymal stem cell-derived exosome-functionalized 3D-Printed hierarchical porous scaffolds with neuro-promotive properties for enhancing innervated bone regeneration. ACS Nano.

[37] Xu Y., Xu C., He L., Zhou J., Chen T., Ouyang L., Guo X., Qu Y., Luo Z., Duan D. (2022). Stratified-structural hydrogel incorporated with magnesium-ion-modified black phosphorus nanosheets for promoting neuro-vascularized bone regeneration. Bioact. Mater..

[38] Gordon T. (2020). Peripheral nerve regeneration and muscle reinnervation. Int. J. Mol. Sci..

[39] Nicoletti V.G., Pajer K., Calcagno D., Pajenda G., Nógrádi A. (2022). The role of metals in the neuroregenerative action of BDNF, GDNF, NGF and other neurotrophic factors. Biomolecules.

[40] Avraham O., Deng P.Y., Jones S., Kuruvilla R., Semenkovich C.F., Klyachko V.A., Cavalli V. (2020). Satellite glial cells promote regenerative growth in sensory neurons. Nat. Commun..

[41] Lezana J.P., Dagan S.Y., Robinson A., Goldstein R.S., Fainzilber M., Bronfman F.C., Bronfman M. (2016). Axonal PPARγ promotes neuronal regeneration after injury. Dev Neurobiol.

[42] Nix P., Hisamoto N., Matsumoto K., Bastiani M. (2011). Axon regeneration requires coordinate activation of p38 and JNK MAPK pathways. Proc. Natl. Acad. Sci. U. S. A..

[43] Mo J., Anastasaki C., Chen Z., Shipman T., Papke J., Yin K., Gutmann D.H., Le L.Q. (2021). Humanized neurofibroma model from induced pluripotent stem cells delineates tumor pathogenesis and developmental origins. J. Clin. Investig..

[44] Duan Q., Zheng H., Qin Y., Yan J., Wang J., Burgess S.M., Fan C. (2024). Stat3 has a different role in axon growth during development than it does in axon regeneration after injury. Mol. Neurobiol..

[45] Liu X., Blazejewski S.M., Bennison S.A., Toyo-Oka K. (2021). Glutathione S-transferase Pi (Gstp) proteins regulate neuritogenesis in the developing cerebral cortex. Hum. Mol. Genet..

[46] Stegen S., Carmeliet G. (2018). The skeletal vascular system - breathing life into bone tissue. Bone.

[47] Filipowska J., Tomaszewski K.A., Niedźwiedzki Ł., Walocha J.A., Niedźwiedzki T. (2017). The role of vasculature in bone development, regeneration and proper systemic functioning. Angiogenesis.

[48] Ma J., Zhao N., Zhu D. (2015). Endothelial cellular responses to biodegradable metal zinc. ACS Biomater. Sci. Eng..

[49] Arnaoutova I., Kleinman H.K. (2010). In vitro angiogenesis: endothelial cell tube formation on gelled basement membrane extract. Nat. Protoc..

[50] Chen H., Hu B., Lv X., Zhu S., Zhen G., Wan M., Jain A., Gao B., Chai Y., Yang M., Wang X., Deng R., Wang L., Cao Y., Ni S., Liu S., Yuan W., Chen H., Dong X., Guan Y., Yang H., Cao X. (2019). Prostaglandin E2 mediates sensory nerve regulation of bone homeostasis. Nat. Commun..

[51] Wu M., Liu H., Li D., Zhu Y., Wu P., Chen Z., Chen F., Chen Y., Deng Z., Cai L. (2024). Smart-responsive multifunctional therapeutic system for improved regenerative microenvironment and accelerated bone regeneration via mild photothermal therapy. Adv. Sci. (Weinh.).

[52] Kohara H., Tajima S., Yamamoto M., Tabata Y. (2010). Angiogenesis induced by controlled release of neuropeptide substance P. Biomaterials.

[53] Tao R., Mi B., Hu Y., Lin S., Xiong Y., Lu X., Panayi A.C., Li G., Liu G. (2023). Hallmarks of peripheral nerve function in bone regeneration. Bone Research.

