# Supplementary material for: Neurofunctional MOF nanoparticles integrated with extracellular matrix hydrogel for neuro-Vascularized bone regeneration
Source: Mater Today Bio. 2025 Nov 12;35:102541. doi: 10.1016/j.mtbio.2025.102541 (PMC12663671; doi:10.1016/j.mtbio.2025.102541)
Supplement: Multimedia component 1 [file mmc1.docx]

**Supporting Information**

**Extracellular Matrix Hydrogels Modified with Nano MOF Encapsulated Substance P for Neurovascular-Driven Bone Regeneration**

Ning Sheng^1,†^, Runze Yang^3,7,†^, Jie Wang^4^, Wenting Wu^2^, Man Zhe^5^, Qing-Yi Zhang^6^, Rong Nie^6^, Long Chen^1^, Fei Xing^2,*^, and Li Sun^1,*^

^1^Department of Orthopedics, Guizhou Provincial People's Hospital, Guiyang 550000, Guizhou, China.

^2^Department of Pediatric Surgery, Division of Orthopedic Surgery, Orthopedic Research Institute, Laboratory of Stem Cell and Tissue Engineering, State Key Laboratory of Biotherapy, West China School of Medicine, West China Hospital, Sichuan University, Chengdu 610041, China.

^3^Department of Orthopedics, Sichuan Provincial People's Hospital, University of Electronic Science and Technology of China, Chengdu, 610072, China

^4^Department of Foot and Ankle Surgery, Honghui Hospital of Xi'an Jiaotong University, Xi'an City, Shaanxi 710054, People's Republic of China

^5^Animal Experiment Center, West China Hospital, Sichuan University, Chengdu 610041, China.

^6^Department of Orthopedic Surgery and Orthopedic Research Institute, Laboratory of Stem Cell and Tissue Engineering, State Key Laboratory of Biotherapy, West China Hospital, Sichuan University, Chengdu 610041, China.

^7^Sports Medicine Center, Department of Orthopedic Surgery/Orthopedic Research Institute, West China Hospital, Sichuan University, Chengdu, Sichuan 610064, China.

^†^These authors contributed equally to this work

^*^ Correspondence should be addressed to:

Fei Xing: Department of Pediatric Surgery, Division of Orthopedic Surgery, Orthopedic Research Institute, Laboratory of Stem Cell and Tissue Engineering, State Key Laboratory of Biotherapy, West China School of Medicine, West China Hospital, Sichuan University, Chengdu 610041. E-mail: xingfeihuaxi@163.com

Li Sun: Department of Orthopedics, Guizhou Provincial People’s Hospital, Guiyang 550000, Guizhou. E-mail: lisun@gzu.edu.cn

1. **Experimental section:**
   1. **Materials**

DMEM, α-MEM, F12/DMEM, penicillin-streptomycin solution (P/S) and fetal bovine serum (FBS) were purchased from Gibco (USA). Trypsinm, phalloidin.-rhodamine, DAPI staining solution and ttriton X-100 were purchased from Sigma (USA). Edta disodium salt dihydrate (EDTA) was purchased from Chron Chemicals (China). EastepTM Super Total RNA Extraction Kit and GoScript™ Reverse Transcription Mix were purchased from Promega (USA). Matrigel matrix was purchased from Corning (USA).

- 1. **Preparation of SIS powder**

First, porcine small intestine was obtained and harvested from healthy pigs and rinsed thoroughly. Then, it was cut into 10 cm sections and washed with PBS, followed by mechanical removal of the tunica serosa and tunica muscularis. Subsequently, tissues were cleaned by continuous washing with PBS and submucosal membranes were immersed in a solution containing methanol and chloroform (1:1, V/V) in a fume hood for 12 h. After incubation, the sample was rinsed with deionized water to remove the organic solvent. Next, the samples were incubated in 0.05% trypsin and 0.05% Edetic Acid for 12 h at 37 ° C, followed by continuous washing with saline solution to remove trypsin. The membrane was then further treated with 0.5% SDS and 0.9% NaCl by shaking the Sodium lauryl sulfate for 4 h. Then rinse thoroughly with a saline solution to remove the detergent. Subsequently, the resulting mixture was freeze-dried for 24 h and ground to a powder using a ball mill. The powder was digested with 0.1% (w/v) pepsin at 37 ° C for 48 h. After freeze-drying, the spongy SIS was ground into SIS powder.

- 1. **Table S1 PCR primers sequence**

| Gene | Primer sequence (5'→3') |
| --- | --- |
| Neurogenesis |  |
| NGF | F: TGATCGGCGTACAGGCAGA  R: GAGGGCTGTGTCAAGGGAAT |
| BDNG | F: GTCAAGTGCCTTTGGAGCCT  R: CTTATGAACCTTTGGAGCCT |
| GDNF | F: CTACGAAACCAAGGAGGAACTGA  R:GGTAAACCAGGCTGTCGTCTAAA |
| GAPDH | F:AGGTCGGTGTGAACGGATTTG  R: TGTAGACCATGTAGTTGAGGTCA |
| Angiogenesis genes |  |
| VEGF | F: TGCGGATCAAACCTCACCA  R: CAGGGATTTTTCTTGTCTTGCT |
| Hif1-α | F: CAAGGCAGCAGAAACCTAC  R: GGAACACCGACAAACCC |
| Notch | F: TCCAACTGCGACACCAAC  R: CAGCGAGCACTCATCCAC |
| GAPDH | F: GGGGCTCTCCAGAACATC  R: TGACACGTTGGCAGTGG |
| Osteogenesis genes |  |
| RUNX2 | F: TCGGAAAGGGACGAGAG  R: TTCAAACGCATACCTGCAT |
| ALP | F: TGATACCTGCCTCACTTCC  R: GAGAGAAACCCACCCTGCT |
| Bglap | F: ACAAGTCCCACACAGCAAC  R: CCAGGTCAGAGAGGCAGA |
| GAPDH | F: CAAATTCCATGGCACCGTCA  R: GACTCCACGACGTACTCAGC |

- 1. **Transcriptomic analysis**

After extracting total RNA from the samples, the RNA that met the quality standards was utilized in the development of the sequencing library. The construction and sequencing of the whole transcriptome libraries were conducted by Gene Denovo Biotechnology Ltd. To ensure the inclusion of all coding RNAs and non-coding RNAs (ncRNAs), an rRNA Removal Kit was employed to eliminate ribosomal RNA. Subsequently, mRNA enrichment was executed. The enriched mRNA was then reverse-transcribed to generate double-stranded cDNA. Following this, cDNA double-end repair and junction addition were performed, followed by PCR amplification to construct the online library and ensure library quality control. Each group comprised four biological replicates. The final ligation products were sequenced using the Illumina HiSeqTM 4000 platform. High-quality clean reads were obtained through the use of FASTP (version 0.18.0), and data de novo assembly was performed using the Trinity short read assembly program. The expression level of the unigene was computed and then normalized to RPKM (reads per kb per million reads). Differential gene expression (DEGs) analysis was carried out using DESeq2 software. Genes meeting the criteria of a false discovery rate (FDR) below 0.05 and an absolute fold change of ≥2 were classified as differentially expressed genes. To assess the reproducibility between samples and exclude outliers, Principal Component Analysis (PCA) and Pearson correlation coefficient analyses were performed. For functional annotation and enrichment analysis, Gene Ontology (GO) enrichment analysis was employed to detect significantly enriched GO terms among the DEGs. Furthermore, KEGG enrichment analysis was utilized to identify metabolic pathways and signal transduction pathways enriched with DEGs.

1. **Results**


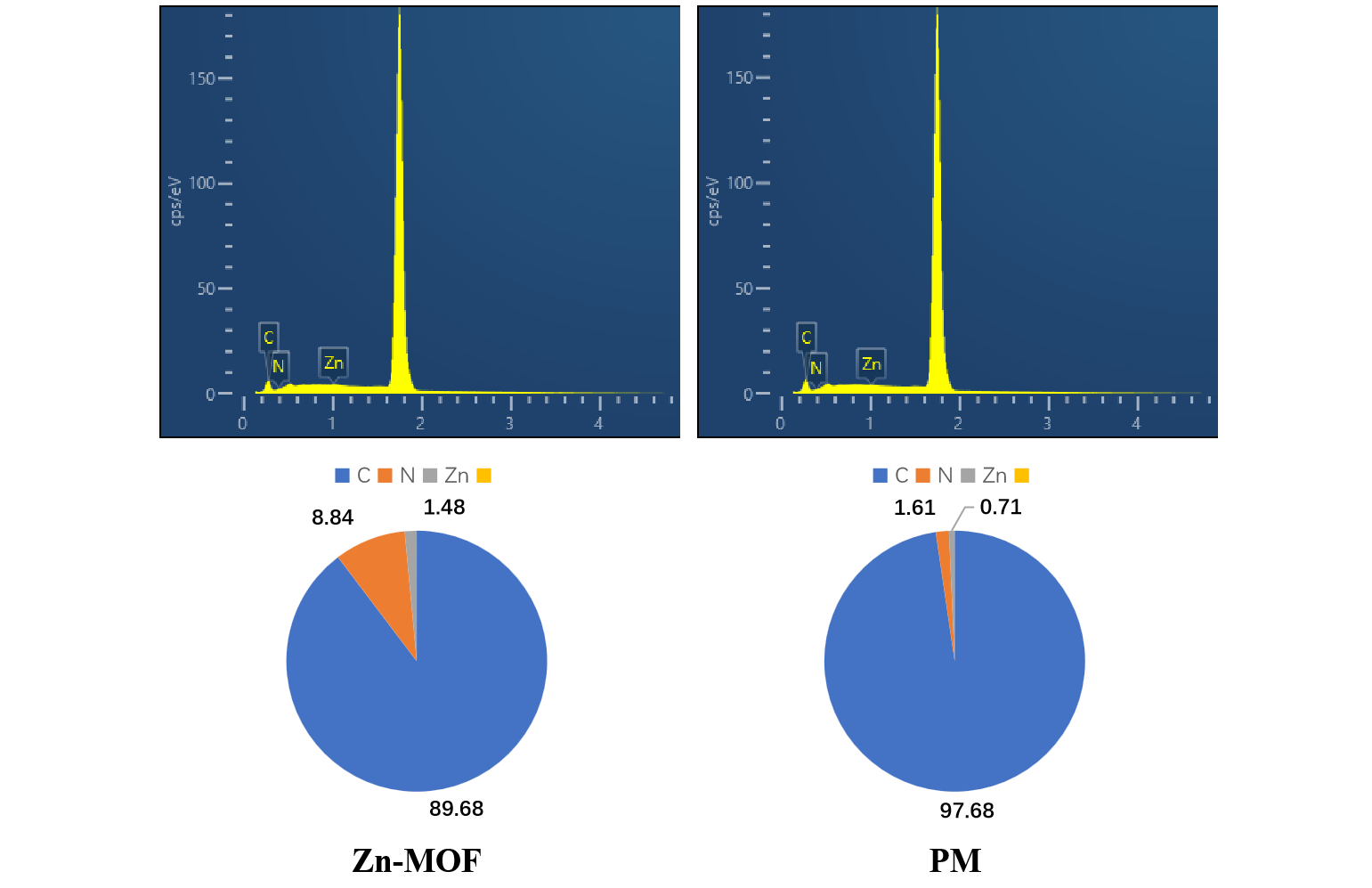


Fig. S1. Energy-dispersive spectroscopy (EDS) images and atomic percent of carbon (C), nitrogen (N), and zinc (Zn).


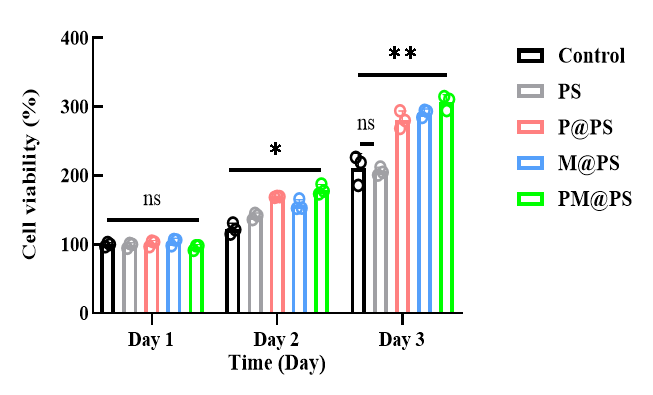


Fig. S2. CCK-8 assay of BMSCs proliferation. (^ns^ *p >* 0.05, ^*^*p* < 0.05, ^**^*p*< 0.01).


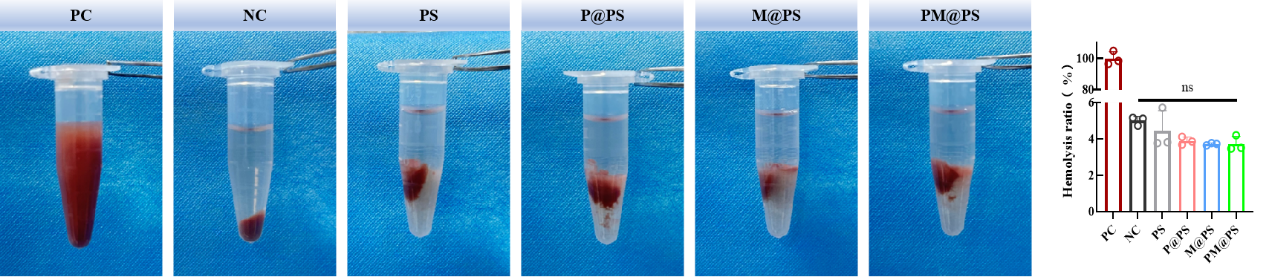


Fig. S3. The hemolysis test of hydrogels. (^ns^ *p >* 0.05).


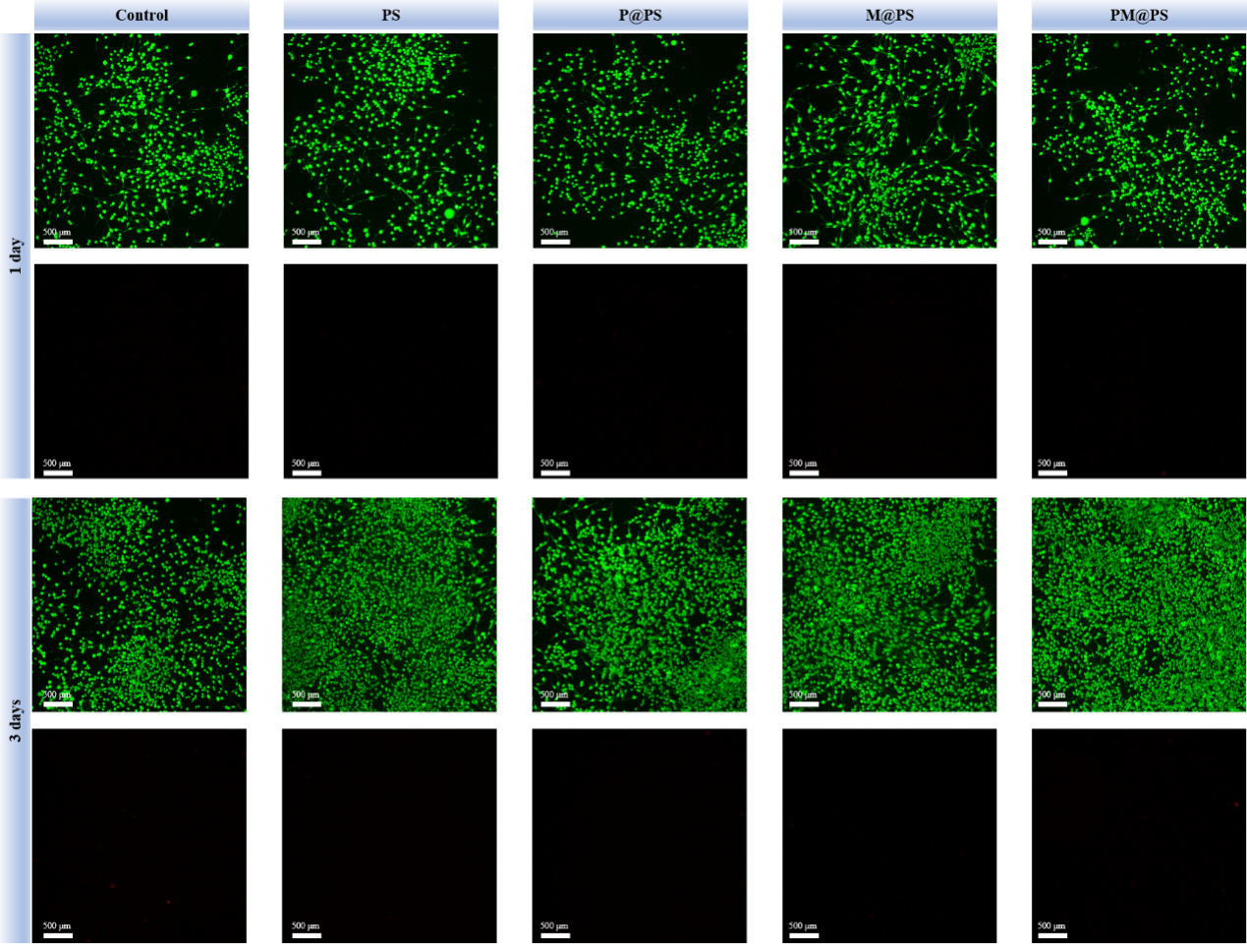
Fig. S4. Live/Dead staining fluorescence images (green means living cells and red means dead cells) of SCs. (Scale bar = 500 μm).


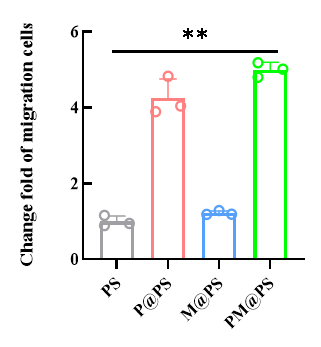


Fig. S5. Semi-quantitative analysis of migration SCs. (^**^*p*< 0.01).


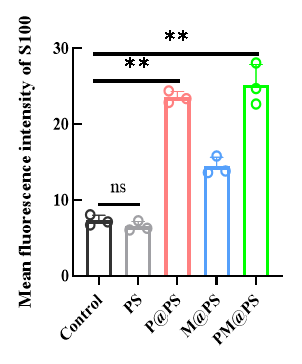


Fig. S6. Semi-quantitative analysis of S100 fluorescence intensity. (^ns^ *p >* 0.05, ^**^*p*< 0.01).


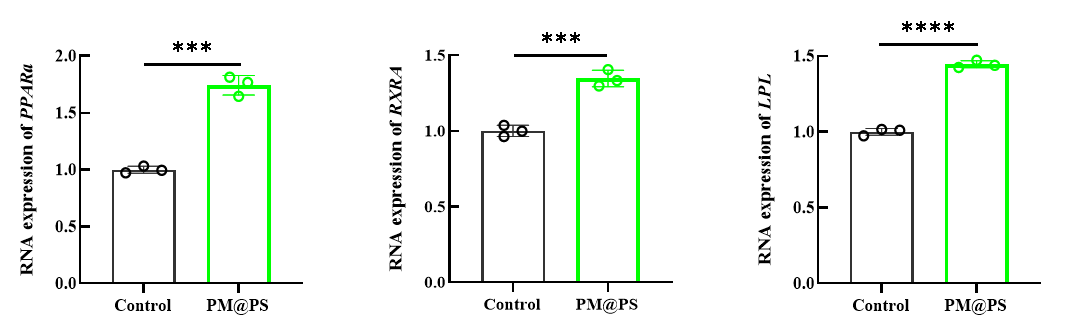


Fig. S7. Genes expression levels of *PPARa, RXRA,* and *LPL* in SCs. (^***^*p*< 0.001, (^****^*p*< 0.0001).


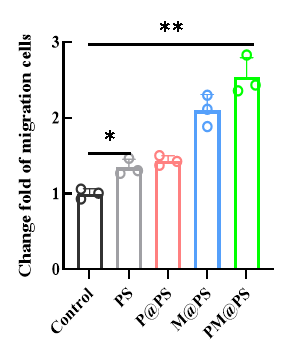


Fig. S8. Semi-quantitative analysis of migration HUVECs. (^*^*p* < 0.05, ^**^*p*< 0.01).


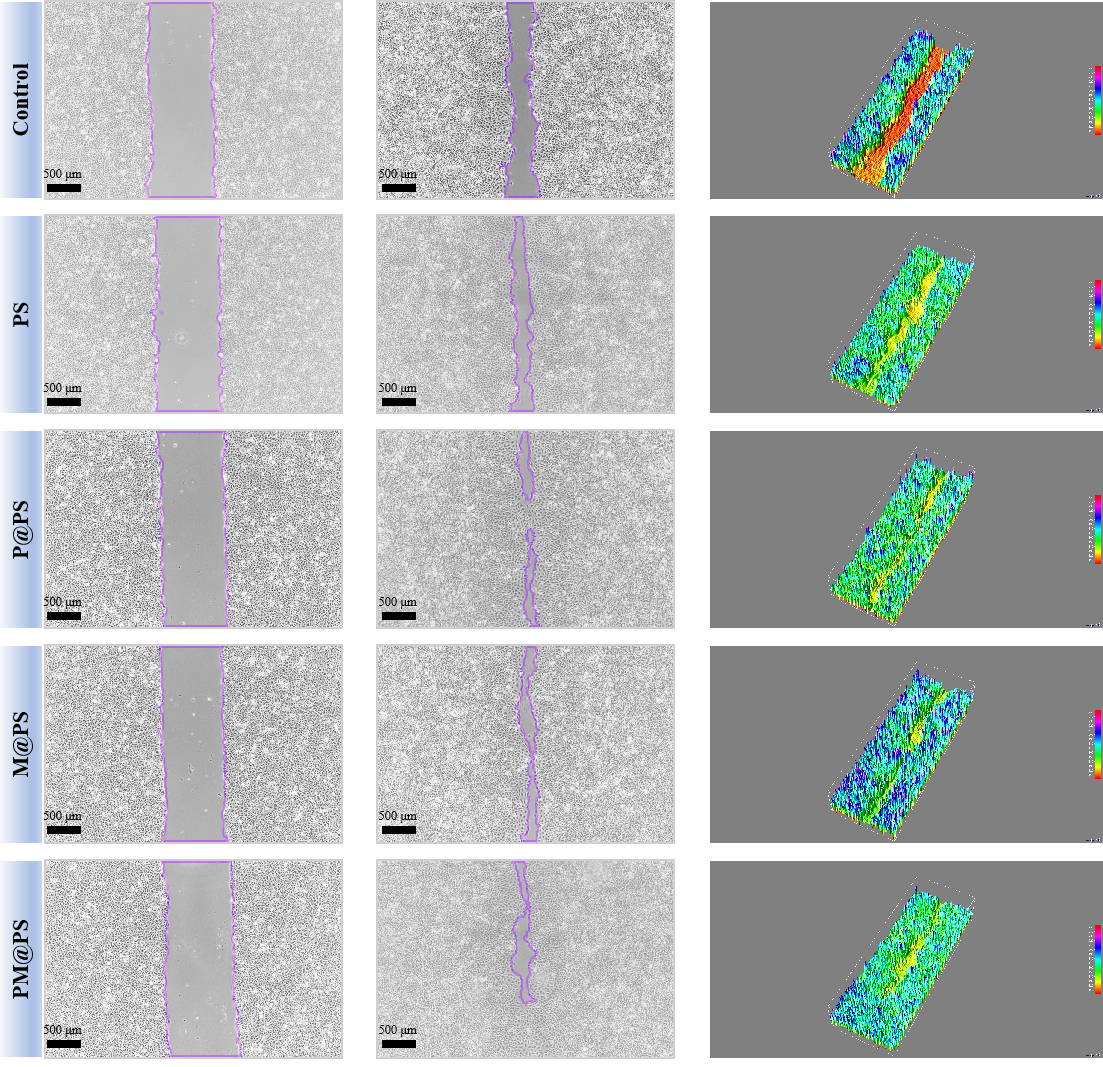


Fig. S9. The scratch wound assay of HUVECs. (Scale bar = 500 μm).


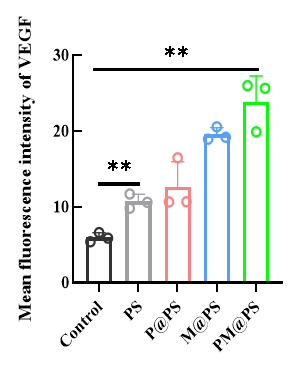


Fig. S10. Semi-quantitative analysis of VEGF fluorescence intensity. (^**^*p*< 0.01).


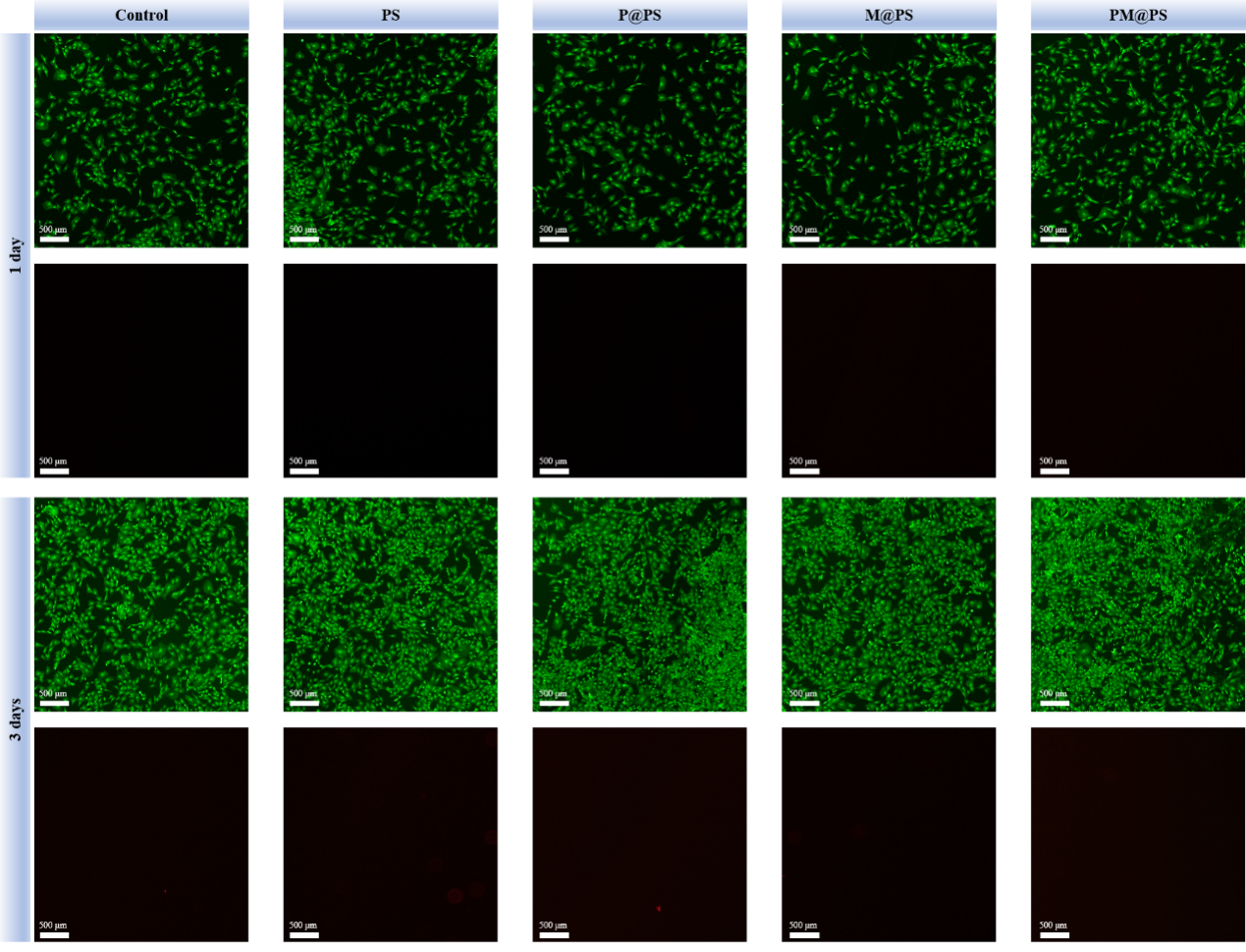


Fig. S11. Live/Dead staining fluorescence images (green means living cells and red means dead cells) of BMSCs. (Scale bar = 500 μm).


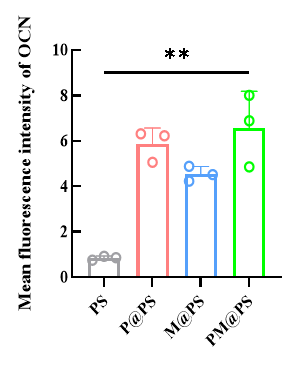


Fig. S12. Semi-quantitative analysis of OCN fluorescence intensity. (^**^*p*< 0.01).


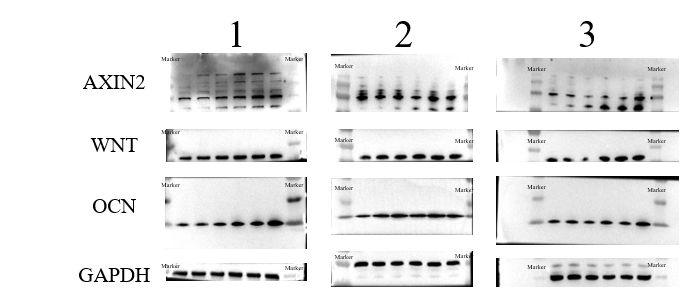


Fig. S13. Original uncut images used for quantitative analysis.


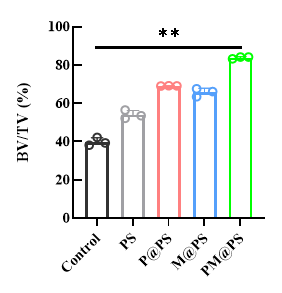


Fig. S14. Statistical analysis of BV/TV based on results of micro-CT. (^**^*p*< 0.01).


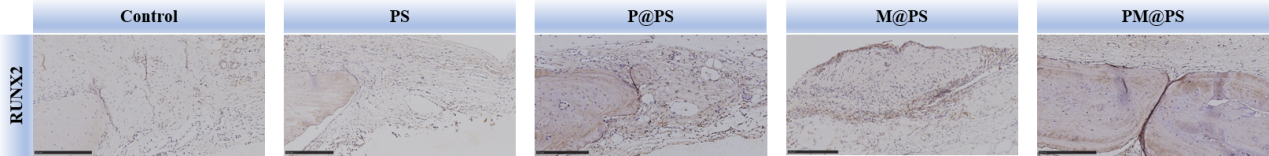


Fig. S15. Immunohistochemical staining of RUNX2 in the defect zones at 12 weeks. (Scale bar = 250 μm).


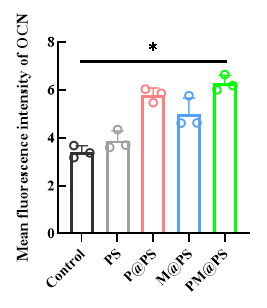


Fig. S16. Semi-quantitative analysis of OCN fluorescence intensity in vivo. (^*^*p*< 0.05).


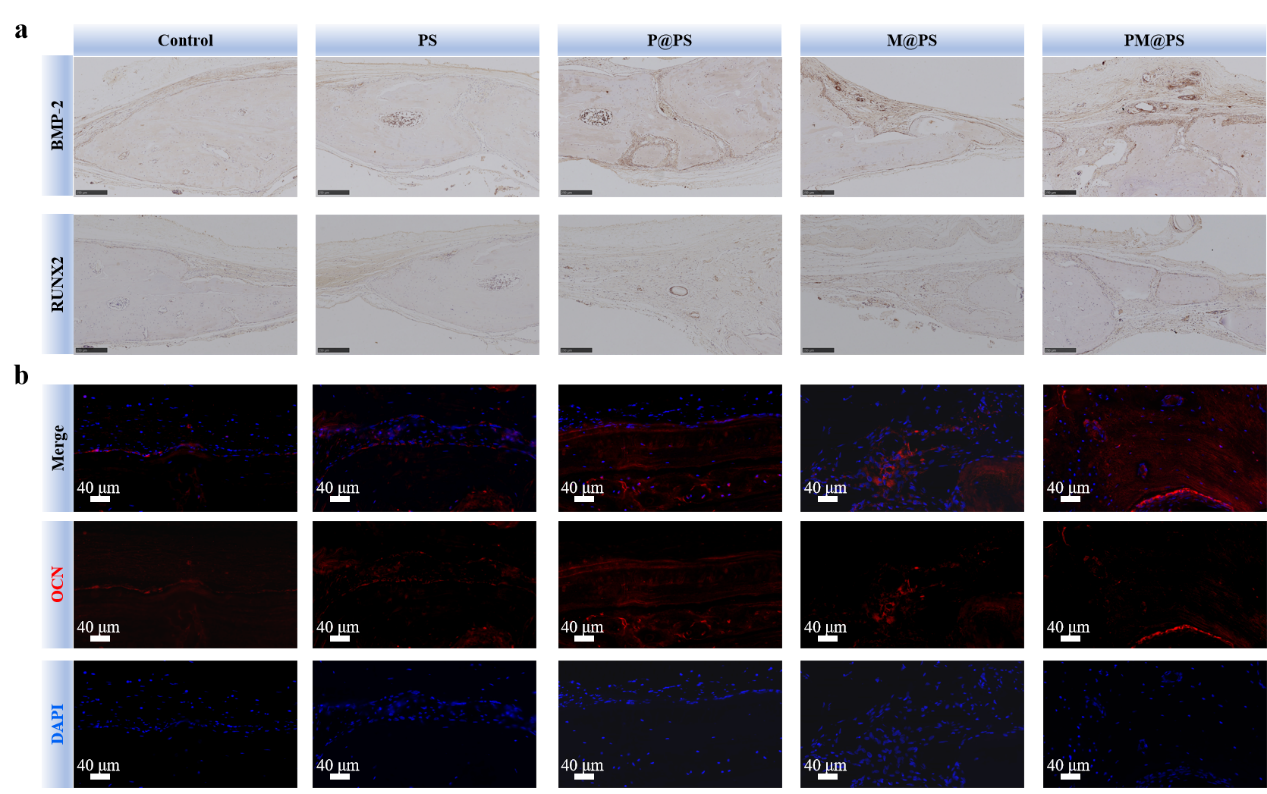


Fig. S17. (a) Immunohistochemical staining of BMP-2 and RUNX2 in the defect zones at 8 weeks (Scale bar = 250 μm); (b) Immunofluorescence staining of OCN (red) in the defect zones at 8 weeks (Scale bar = 40 μm).


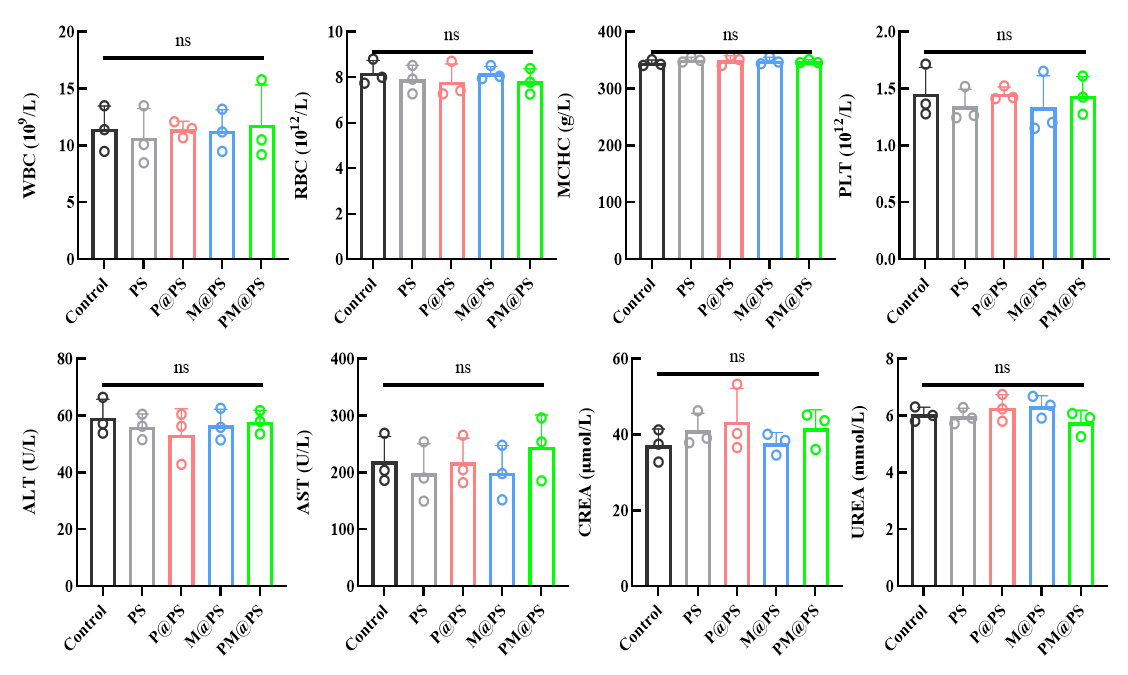


Fig. S18. The routine blood tests (white blood cell, WBC; red blood cell, RBC; mean corpuscular hemoglobin concentration, MCHC; platelet, PLT) and biochemistry assays (glutamic pyruvic transaminase, ALT; aspartate transaminase, AST; creatinine. CREA; urea, UREA) of the hydrogel-implanted rats at 12 weeks. (^ns^ *p >* 0.05).
